# Autophagy facilitates age-related cell apoptosis—a new insight from senile cataract

**DOI:** 10.1038/s41419-021-04489-8

**Published:** 2022-01-10

**Authors:** Jiani Huang, Wangshu Yu, Qin He, Xiaoying He, Ming Yang, Wei Chen, Wei Han

**Affiliations:** 1 Eye Center of the Second Affiliated Hospital, School of Medicine, Zhejiang University, Hangzhou, Zhejiang 310003 PR China; 2 Department of Ophthalmology, The First Affiliated Hospital, School of Medicine, Zhejiang University, Hangzhou, Zhejiang 310003 PR China; 3 Institute of Immunology, School of Medicine, Zhejiang University, Hangzhou, Zhejiang 310058 PR China

**Keywords:** Macroautophagy, Apoptosis, Senescence, Diseases

## Abstract

Age-related cell loss underpins many senescence-associated diseases. Apoptosis of lens epithelial cells (LECs) is the important cellular basis of senile cataract resulted from prolonged exposure to oxidative stress, although the specific mechanisms remain elusive. Our data indicated the concomitance of high autophagy activity, low SQSTM1/p62 protein level and apoptosis in the same LEC from senile cataract patients. Meanwhile, in primary cultured LECs model, more durable autophagy activation and more obvious p62 degradation under oxidative stress were observed in LECs from elder healthy donors, compared with that from young healthy donors. Using autophagy-deficiency HLE-B3 cell line, autophagy adaptor p62 was identified as the critical scaffold protein sustaining the pro-survival signaling PKCι-IKK-NF-κB cascades, which antagonized the pro-apoptotic signaling. Moreover, the pharmacological inhibitor of autophagy, 3-MA, significantly inhibited p62 degradation and rescued oxidative stress-induced apoptosis in elder LECs. Collectively, this study demonstrated that durable activation of autophagy promoted age-related cell death in LECs. Our work contributes to better understanding the pathogenesis of senescence-associated diseases.

## Introduction

With rapid increase of ageing populations, senescence-associated diseases have become the greatest socioeconomic challenge for the next decades. Senescence-associated diseases are characterized by the continuous cell loss and progressive deterioration of organs’ function [1, 2]. Oxidative stress is regarded as the primary etiologic factor for ageing process, which causes damage of biomacromolecules, such as proteins, DNA, and lipids, and ultimately leads to cell senescence and loss [3]. Consequently, the age-related cell loss in postmitotic tissues can contribute to the occurrence of most senescence-associated degenerative diseases, including neurodegenerative Alzheimer’s or Parkinson’s disease [4], cardiomyopathy [5, 6], osteoarthritis [7], and senile cataract [8]. However, the detailed molecular mechanism for age-related cell loss is not yet fully understood.

Senile cataract (also called age-related cataract), a typical senescence-associated ocular disease, is the primary cause of blindness worldwide. Senile cataract usually occurs in elderly people over 50 years of age and is the result of gradual opacification of the lens, an important part of the eye’s optical system [9] (Fig. S1). The lens epithelial cells (LECs), the only type cell in the lens, play a key role in maintaining homeostasis of the lens internal environment, which is crucial for its optical transparency [10]. The previous studies in neurology have observed moderate neuronal loss in the brain of elderly individuals, while neuronal cell number significantly declines in the setting of neurodegenerative disease [11, 12]. Similarly, there is an age-related decrease trend in the cell density of human LECs [13], with a loss of 14% of the total LECs in a 75-year life span [14]. During this process, oxidative stress exacerbates the loss of LECs, and once the cell loss reaches a certain level, senile cataract is caused [10, 15]. Although the exact pathogenesis for senile cataract formation is still far from being fully elucidated, the malfunction of LECs, like oxidative stress-induced apoptosis, is thought to be central for senile cataractogenesis [8, 10, 15, 16].

Macroautophagy (hereafter referred to as autophagy), an evolutionarily conserved self-degradation process in eukaryotic cells, was recently emphasized as a central mechanism for cellular homeostasis and adaptation to stress [17, 18]. Particularly, the relationship between autophagy and human ageing is a cutting-edge topic and remains complex. Many studies found that autophagy declines with age, while elevation of basal autophagic activity can delay cellular senescence and extend the health span of mice [19, 20]. However, some studies hold the opposite opinion that activation of autophagy may facilitate the ageing process. Oncogene-induced senescence was found to be well dependent on prior induction of autophagy, as genetic silencing of autophagy significantly delayed the senescence response [21, 22]. Accumulating evidence further supports that autophagy contributes to the induction of senescence in various tissues and cells, while blockage of autophagy attenuates oxidative stress and DNA damage-induced senescence [23–25]. In line with the latter opinion, the most important autophagy adaptor SQSTM1/p62, which is degraded with cargoes in autolysosomes, has been proved to promote mouse longevity by delaying senescence [26, 27]. Importantly, the protein level of p62 rapidly declines in aged tissues, and loss of p62 is associated with senescence-associated diseases [27–29]. Together, these evidence suggested that autophagy might be a bidirectional regulator of cell senescence, depending on the different stress stimulus and cell types. However, the specific role of autophagy in age-related cell loss is still poorly understood.

In this study, we found the concomitance of high autophagy activity, low SQSTM1/p62 protein level and apoptosis in the same LEC from senile cataract patients. Our data further demonstrated the more durable activation of autophagy triggered by oxidative stress in senescent LECs compared with that in young LECs. Consequently, the excessive degradation of p62 was caused and senescent LECs underwent apoptosis, as p62 was critical to sustaining the pro-survival signaling via the PKCι-IKK-NF-κB cascade.

## Results

### The concomitance of increased autophagy activity and apoptosis in the same LECs from senile cataract patients

**Fig. 1:**
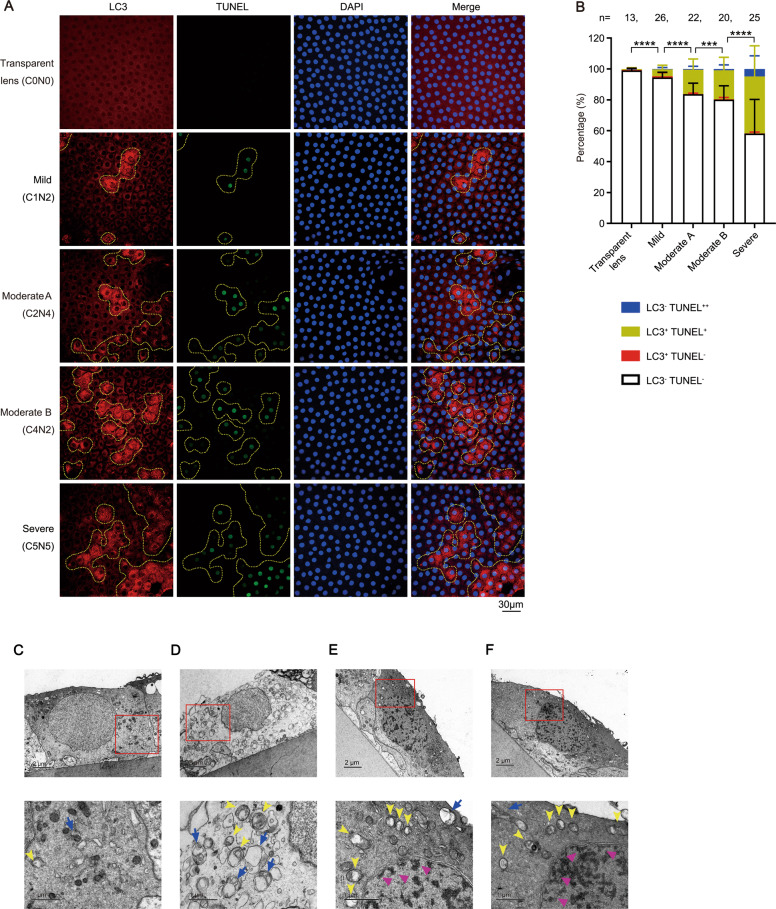
The concomitance of increased autophagy activity and apoptosis in the same LECs from cataract patients. **A** Representative confocal images of LECs from 93 patients with different grade senile cataract and control LECs from 13 patients with a transparent lens. LECs are stained with LC3 antibody (autophagosome, red), TUNEL labeling (DNA fragment, green), and DAPI (nucleus, blue). Yellow dotted irregular circles indicate cells with LC3 puncta. Scale bar, 30 μm. **B** Grouped stacked bars denoting the percentages of LC3^-^TUNEL^-^, LC3^+^TUNEL^-^, LC3^+^TUNEL^+^ and LC3^-^TUNEL^++^ LECs in lens capsules from patients as shown in **A**. Bars represent mean ± SD. *n* shows the numbers of patients analyzed. ***p < 0.001, ****p < 0.0001 (*χ*^2^ test followed by Bonferroni post hoc test). **C**–**F** Representative electron microscopic images of LECs from ten patients with moderate to severe senile cataract (lower panels, expanded view of the red line boxed areas in upper panel; yellow arrowheads, autophagosomes with double membranes; blue arrows, autophagolysosomes with single membrane; magenta triangles, condensed and edged chromatin).

To explore the role of autophagy in the pathological process of senile cataract, lens capsule specimens from senile cataract patients in different grades were collected (Table S1). The percentage of apoptotic LECs in lens capsules was significantly increased with the aggravation of senile cataract grade (Fig. 1A, B). The most striking results were that all cells with a weak TUNEL signal (TUNEL^+^, early stage of apoptosis) were always concomitant with elevated autophagic activity (clear LC3 puncta, LC3^+^), while LC3^+^ TUNEL^−^ cells were occasionally observed, indicating the remarkable concomitance of increased autophagy activity and apoptosis in the same LEC (Fig. 1A, B). Moreover, there were small patches which comprised a few to dozens LECs with a strong TUNEL signal (TUNEL^++^) in lens capsules from patients (Fig. S2). These TUNEL^++^ LECs are thought to be apoptotic cells that have existed for long time due to the absence of macrophages in lens tissue [30].

Lens capsules from patients were further evaluated using transmission electron microscopy. As expected, most of non-apoptotic LECs contained few autophagosomes and autolysosomes (Fig. 1C). Besides, there were two other types of LEC: (1) a few non-apoptotic LECs showed markedly elevated autophagy (Fig. 1D); and (2) some apoptotic cells showed markedly elevated autophagy (Fig. 1E, F).

### Autophagy promotes cell senescence and age-related cell death in human LECs

**Fig. 2:**
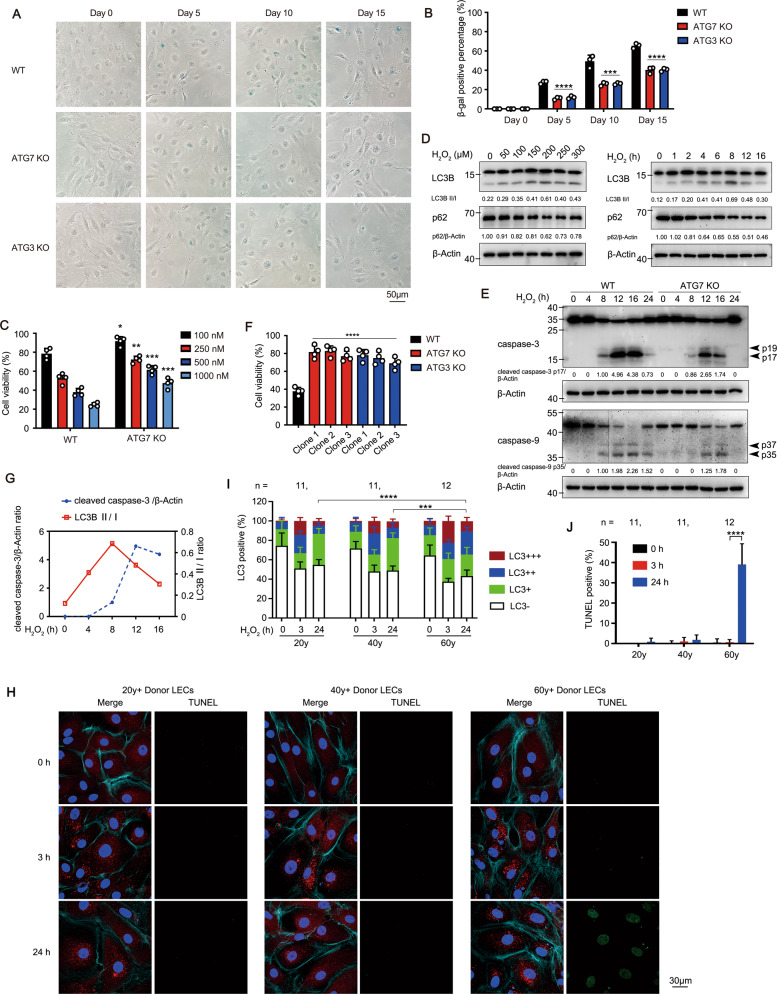
Autophagy promotes cell senescence and age-related cell death in human LECs. **A** β-gal staining of WT, ATG7 KO and ATG3 KO cells at indicated days post 20 μM H_2_O_2_ treatment was imaged by microscopy. Scale bar, 50 μm. **B** Quantification of the percentages of cells with β-gal positive staining treated as in (A). Data are mean ± SD from three random fields; ***p < 0.001, ****p < 0.0001, KO vs WT cells in the same time point (one-way ANOVA followed by Bonferroni post hoc test). **C** Viability of WT and ATG7 KO HLE-B3 cells determined by CCK8 assay. Cells were pretreated with 20 μM H_2_O_2_ for 5 days, then exposed to different concentrations of rapamycin for another 24 h. Mean ± SD from four independent experiments; *p < 0.05, **p < 0.01, ***p < 0.001, KO *vs* WT cells at the same dosage of rapamycin (unpaired Student’s *t* test). **D** Immunoblots showing LC3B and p62 levels in HLE-B3 cells stimulated with different concentrations of H_2_O_2_ for 8 h (left panel) or with 200 μM H_2_O_2_ for indicated times (right panel). **E** Immunoblots showing activated caspases (arrowheads) in WT and ATG7 KO HLE-B3 cells exposed to 200 μM H_2_O_2_ for indicated times. **F** Viability of ATG7 KO and ATG3 KO HLE-B3 cells stimulated with 200 μM H_2_O_2_ for 24 h determined by CCK8 assay. Mean ± SD from four independent experiments; ****p < 0.0001 (one-way ANOVA followed by Bonferroni post hoc test). **G** Quantitative analysis of caspase-3 activation as in **E** and LC3B II/I conversion as in **D** during 200 μM H_2_O_2_ challenge. **H** Representative confocal images of primary cultured human LECs from 34 healthy donors of different age groups stimulated with 50 μM H_2_O_2_ for indicated times. LECs are stained with LC3 antibody (red), TUNEL labeling (green), DAPI (blue), and Phalloidin-iFluor 647 (F-Actin, cyan). Scale bar, 30 μm. **I** Grouped stacked bars showing the percentages of LC3^-^, LC3^+^, LC3^+ +^, and LC3^+++^ in primary cultured human LECs from healthy donors treated as in **H** (mean ± SD; *n* = numbers of healthy donors analyzed; ***p < 0.001, ****p < 0.0001, Kruskal-Wallis test followed by Bonferroni post hoc test). **J** Percentages of TUNEL-positive primary cultured human LECs from healthy donors treated as in **H** (mean ± SD; *n* = numbers of healthy donors analyzed; ****p < 0.0001, one-way ANOVA followed by Bonferroni post hoc test).

The role of autophagy in ageing process of human LECs was investigated, including cell senescence and loss. Firstly, whether autophagy promotes the cellular senescence of LECs was tested. In the immortalized human LECs line HLE-B3, sustained stimulation of low dose rapamycin, an autophagy inducer, led to the increasing senescence-associated β-galactosidase staining (Fig. S3A–C). In the oxidative stress stimulus model, low dose (≤50 μM) hydrogen peroxide (H_2_O_2_) triggered lasting activation of autophagy and premature senescence of HLE-B3 cells (Fig. S3D–F). Moreover, ablation of different autophagy-related genes (ATG) via the CRISPR/Cas9 system was used to rule out the non-autophagic function of ATG proteins (Fig. S3G). Both ATG7 and ATG3 knockout (KO) in HLE-B3 cells obviously delayed the oxidative stress-induced senescence (Fig. 2A, B).

Secondly, we evaluated whether autophagy facilitates cell death in the senescent LECs. HLE-B3 cells were thought to enter pre-senescent state after being stimulated with 20 μM H_2_O_2_ for 5 days in our system, and then treated with rapamycin to further elevate autophagy activity. Under rapamycin stimulation, the wild-type (WT) cells showed increased sensitivity to apoptosis in a dose-dependent pattern, whereas the ATG7 KO cells exhibited resistance to apoptosis (Fig. 2C and Fig. S4A). To further assess the effect of autophagy blockage on cell death, we used the optimized 200 μM to trigger the highest autophagy activity, obvious apoptosis and casapase-3 activation in HLE-B3 cells (Fig. 2D, E and Fig. S4B). The autophagy-deficient cells exhibited resistance to H_2_O_2_-induced apoptosis and lower cleavage level of caspase-3/−9 compared with the controls (Fig. 2E, F and Fig. S4C, D). Importantly, the peaks of LC3I/II conversion (8 h) and caspase-3 activation (12 h) appeared consecutively in WT cells during oxidative stress challenge, confirming autophagy preceding apoptosis (Fig. 2G). Meanwhile, ultraviolet (UV) radiation, another oxidative stress, also triggered higher autophgic level and apoptosis ratio in HLE-B3, whereas knockout of ATG7/ATG3 rescued apoptosis (Fig. S4E, F). Of note, H_2_O_2_ induces multiple modalities of cell death including apoptosis, necroptosis, pyroptosis, ferroptosis, and oxeiptosis [31–34]. In our experiments, pretreatment with Z-VAD (the pan-caspase inhibitor, not inhibitors for other type cell death) almost completely rescued cell viability and caspase-3 cleavage in WT cells (Fig. S4G, H), further validating the apoptotic cell death of HLE-B3 under oxidative stress.

To investigate whether human LECs from elder healthy individuals were more sensitive to oxidative stress-induced apoptosis compared with the younger individuals, lens capsules from different age donors, young (20–30 years), middle-aged (40–50 years), and old (60–70 years), were cultured (Table S1). The numbers of LC3 puncta in three groups were significantly increased after stimulation with 50 μM H_2_O_2_ for 3 h (Fig. 2H, I). Interestingly, at 24 h after stimulation, most LC3 puncta in young and middle-aged LECs were obviously dissipated, whereas the number of LC3 puncta in old LECs was still remained at a high level, with some showing TUNEL-positive staining (Fig. 2H–J). These data indicated that oxidative stress induced more durable activation of autophagy, which may contribute to the sensitivity to apoptosis in senescent LECs.

To rule out the effect of autophagy on cell proliferation, we verified that ablation of ATG7 gene did not alter the cell cycle process in HLE-B3 cells challenged by H_2_O_2_ (Fig. S5A, B). To rule out the possibility that the elevation of autophagy may be due to blockade of late-stage autophagy, we confirmed that the numbers and areas of GFP-LC3 puncta in primary cultured LECs from GFP-LC3 transgenic mice were significantly increased after exposure to H_2_O_2_, while puncta were further elevated when bafilomycin (Baf) A1, an inhibitor of late-stage autophagy, was added (Fig. S6A, B), indicating increases autophagic flux in LECs.

### Autophagy facilitates apoptosis of LECs independent of ROS

**Fig. 3:**
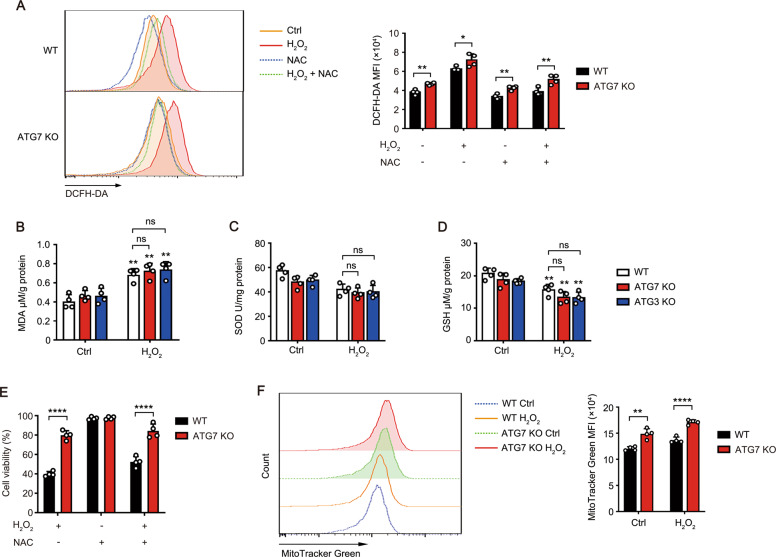
Autophagy facilitates apoptosis of LECs independent of ROS. **A** Representative ROS levels in WT and ATG7 KO HLE-B3 cells pretreated with 10 mM NAC for 1 h, followed by 200 μM H_2_O_2_ stimulation for 24 h assessed by flow cytometry using the ROS sensor DCFH-DA (left). Right panel, quantitative data from four independent experiments (mean ± SD; *p < 0.05, **p < 0.01, unpaired Student’s *t* test). MDA (**B**), SOD (**C**), and GSH (**D**) levels in WT, ATG7, or ATG3 KO HLE-B3 cells with or without 200 μM H_2_O_2_ exposure for 24 h measured by respective kits (MDA level determined by TBRAS assay and normalized to protein concentration). Quantitative data are presented as mean ± SD from four independent experiments (**p < 0.01, H_2_O_2_-treated vs control group; two-way ANOVA followed by Bonferroni post hoc test). **E** Viability of WT and ATG7 KO HLE-B3 cells pretreated with or without 10 mM NAC for 1 h, followed by 200 μM H_2_O_2_ stimulation for 24 h determined by CCK8 assay. Mean ± SD from four independent experiments; ****p < 0.0001 (unpaired Student’s *t* test). **F** MitoTracker labeling and FACS analysis of mitochondrial mass in WT and ATG7 KO HLE-B3 cells with or without 200 μM H_2_O_2_ exposure for 24 h. Right panel, quantitative data from four independent experiments (mean ± SD; **p < 0.01, ****p < 0.0001, unpaired Student’s *t* test).

Chronic exposure to oxidative stress results in excessive accumulation of intracellular reactive oxygen species (ROS), which may consequently induce LECs apoptosis [10, 35]. The ROS level in HLE-B3 cells was significantly increased 24 h after exposure to H_2_O_2_, while ATG7 KO cells showed moderately higher level of ROS than WT cells (Fig. 3A). The levels of lipid peroxidation product malondialdehyde (MDA) were increased both in WT and KO cells similarly (Fig. 3B). Accordingly, the levels of antioxidant enzyme superoxide dismutases (SOD) and antioxidant agents reduced glutathione (GSH) were both decreased, and showed no difference between WT and KO cells (Fig. 3C, D). Pretreatment with N-acety-L-cysteine (NAC) restored ROS level to baseline (Fig. 3A), whereas ATG7 KO still exhibited stronger resistance to H_2_O_2_-induced apoptosis than WT cells (Fig. 3E). Combined with the evidence of lower percentage of apoptosis and higher ROS level in autophagy-deficient cells than that in WT cells, one can speculate that the pro-apoptotic activity of autophagy is ROS independent.

The mitochondrion is an important source of endogenous ROS. Excessive ROS can reciprocally disturb the cellular homeostasis and induce mitophagy [36]. We found that autophagy-deficient cells had a greater mitochondrial mass than WT cells with or without H_2_O_2_ stimulation, suggesting the potential involvement of mitophagy in the control of intracellular ROS levels (Fig. 3F).

### Autophagy facilitates apoptosis through inhibiting the NF-κB pathway in LECs

**Fig. 4:**
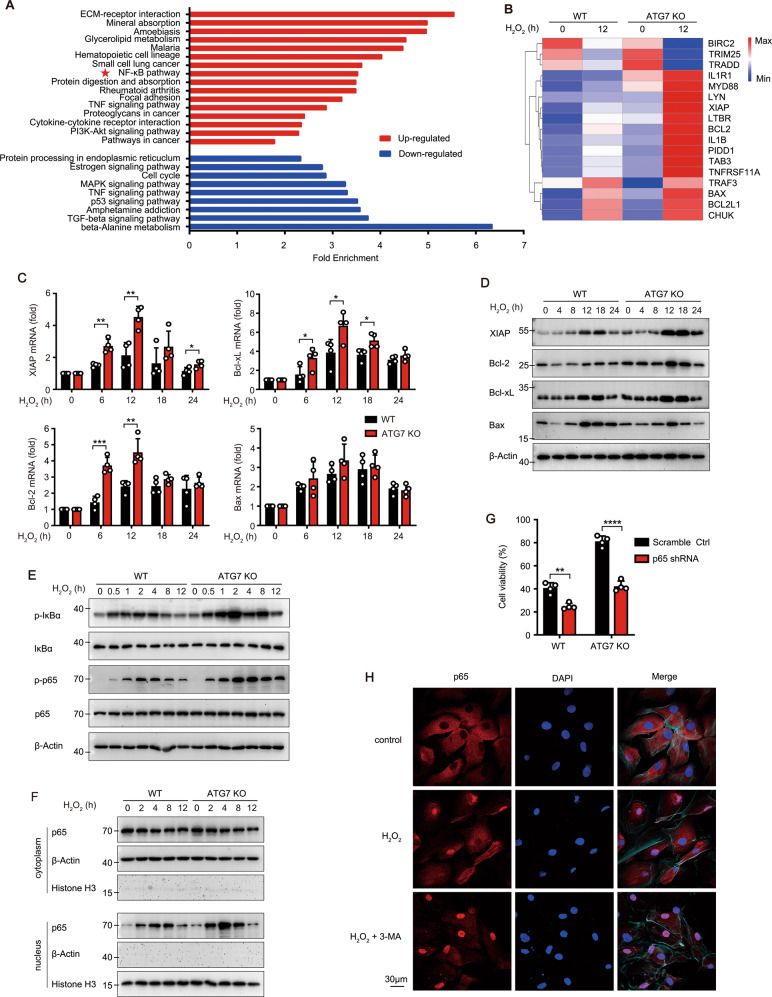
Autophagy facilitates apoptosis through inhibiting the NF-κB pathway in LECs. **A** Fold-enrichment of significantly changed pathways (*p* < 0.05) between H_2_O_2_-treated ATG7 KO *vs* H_2_O_2_-treated WT HLE-B3 cells based on KEGG (Kyoto Encyclopedia of Genes and Genomes)-enrichment analysis. (red, up-regulated pathways; blue, down-regulated pathways). **B** Heatmap of differentially expressed genes in the NF-κB pathway *via* RNA-seq. RNA expression profiles from ATG7 KO and WT HLE-B3 cells with or without 200 μM H_2_O_2_ exposure for 12 h. **C** Relative mRNA levels of XIAP, Bcl-2, Bcl-xL, and Bax in WT and ATG7 KO HLE-B3 cells stimulated with 200 μM H_2_O_2_ for indicated times detected by real-time PCR (mRNA levels normalized by that in cells without H_2_O_2_ treatment). **D** Immunoblots showing protein levels of XIAP, Bcl-2, Bcl-xL, and Bax in WT and ATG7 KO HLE-B3 cells exposed to 200 μM H_2_O_2_ for indicated times. **E** Immunoblots showing total NF-κB p65, phosphorylated p65, total IκBα, and phosphorylated IκBα levels in WT and ATG7 KO HLE-B3 cells exposed to 200 μM H_2_O_2_ for indicated times. **F** Immunoblots showing protein level of p65 in nuclear and cytosolic extracts of WT and ATG7 KO HLE-B3 cells exposed to 200 μM H_2_O_2_ for indicated times (Histone H3 is the internal control for nucleus protein). **G** Viability of WT and ATG7 KO HLE-B3 cells after knockdown of p65 expression exposed to 200 μM H_2_O_2_ for 24 h. **H** Confocal images of p65 (red) nuclear translocation in primary cultured human LECs from five elder healthy donors pretreated of 0.5 mM 3-MA for 3 h, followed by 50 μM H_2_O_2_ exposure for another 1 h. Nucleus stained with DAPI (blue) and F-Actin stained with Phalloidin-iFluor 647 (cyan) to show the cell outline. Scale bar, 30 μm. All quantitative data are presented as mean ± SD from four independent experiments (*p < 0.05, **p < 0.01, ***p < *0.001*, ****p < 0.0001, unpaired Student’s *t* test).

To investigate the potential signalling pathway responsible for autophagy-facilitating apoptosis, RNA-Seq analysis was performed in ATG7 KO and WT HLE-B3 cells with or without H_2_O_2_ stimulation. One intriguing result was that ATG7 KO in HLE-B3 cells with H_2_O_2_ stimulation resulted in significant upregulation of NF-κB pathway, a pro-survival signaling pathway, and increased expression level of the NF-κB target genes, including XIAP, Bcl-2, Bcl-xL, and Bax (Fig. 4A, B). Indeed, the mRNA and protein levels of anti-apoptotic proteins (XIAP, Bcl-2, and Bcl-xL) were obviously higher in ATG7 KO cells than in WT cells, while expression of the pro-apoptotic protein Bax remained unchanged (Fig. 4C, D). Moreover, the phosphorylation of NF-κB p65 and IκB as well as translocation of p65 to the nucleus in ATG7 KO cells was significantly higher and more durable than in WT cells (Fig. 4E, F). As expected, knockdown of p65 with two different RNAi sequences both significantly restored the sensitivity of ATG7 KO cells to H_2_O_2_-induced apoptosis, indicating that the autophagy-facilitating apoptosis was dependent on NF-κB pathway (Figs. S7A and 4G). Meanwhile, ATG3 KO in HLE-B3 cells and treatment of 3-MA or Baf A1 also resulted in enhanced p65 activation and cell viability (Fig. 2F and S8A–D). Conversely, the autophagy inducer rapamycin significantly decreased p65 phosphorylation and cell viability (Fig. S8B, E).

Notably, in primary cultured LECs from elderly healthy donors (60–70 years), 50 μM H_2_O_2_ also triggered the partial nuclear translocation of p65, while pretreatment with 3-MA further enhanced the p65 translocation level (Fig. 4H). Collectively, these data suggested the important role of NF-κB signaling in autophagy-facilitating apoptosis.

### Autophagy selectively degrades p62 protein in LECs

**Fig. 5:**
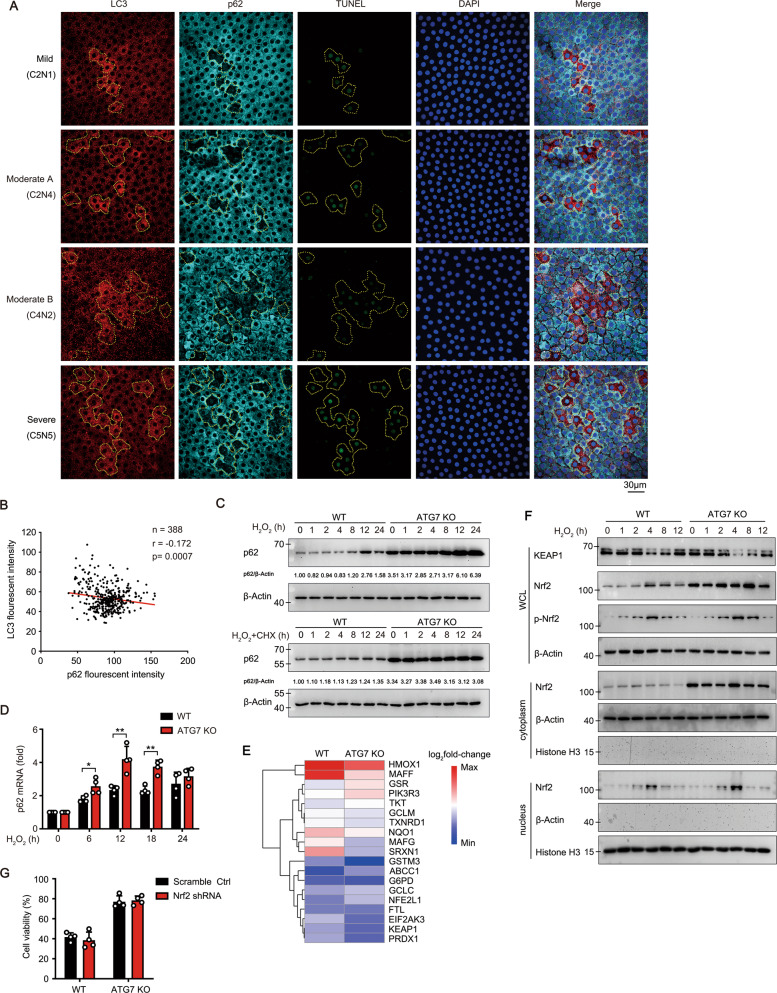
Autophagy selectively degrades p62 protein in LECs. **A** Representative confocal images of LECs from 18 patients with different grades senile cataract stained with LC3 antibody (red), p62 antibody (cyan), TUNEL labeling (green), and DAPI (blue). The laser intensity and pinhole were fixed for all samples. Yellow dotted irregular circles indicate cells with LC3 puncta. Scale bar, 30 μm. **B** Correlation between p62 fluorescence intensity and LC3 fluorescence intensity in LECs from patients with different grades senile cataract stained as in **A** (More than 380 cells from four groups were analyzed; each symbol represents one cell). *p* = 0.0007 by Pearson’s correlation analysis. **C** Immunoblots showing the protein levels of p62 in WT and ATG7 KO HLE-B3 cells stimulated with 200 μM H_2_O_2_ for indicated times, in the presence or absence of 100 μM CHX. **D** Real-time PCR results for the relative mRNA levels of p62 in WT and ATG7 KO HLE-B3 cells stimulated with 200 μM H_2_O_2_ for indicated times. The mRNA levels are normalized by those in cells without H_2_O_2_ treatment. **E** Heatmap of expression levels (log_2_fold-change) of representative Nrf2 pathway genes in ATG7 KO and WT HLE-B3 cells. Data show up-regulated (red) or down-regulated (blue) gene expressions after treated with 200 μM H_2_O_2_ compared with untreated. **F** Immunoblots showing protein level of Nrf2 in whole-cell lysates (WCL), nuclear and cytosolic extracts of WT and ATG7 KO HLE-B3 cells exposed to 200 μM H_2_O_2_ for indicated times. **G** Viability of WT and ATG7 KO HLE-B3 cells after knockdown of Nrf2 expression exposed to 200 μM H_2_O_2_ for 24 h. Quantitative data are presented as mean ± SD from four independent experiments (*p < 0.05, **p < 0.01, unpaired Student’s *t* test).

SQSTM1/p62 is the main adaptor of selective autophagy and act as a multifunctional scaffold protein to control the activation of some key proteins, such as NF-κB, Nrf2, and mTORC1. Given the importance of p62-NF-κB pathway in cell survival and tumorigenesis, we further investigated the degradation of p62 protein in LECs from senile cataract patients. Interestingly, the protein level of p62 showed a significant negative correlation with autophagic activity (LC3 puncta), as evidenced by the pattern that the almost complete absence of p62 protein in some LECs was always concomitant with the high autophagic activity and weak TUNEL signal (Fig. 5A, B). This finding indicates the potential role of p62 in the cell fate of LECs. In H_2_O_2_-treated HLE-B3 cells, the protein and mRNA levels of p62 were both significantly increased in ATG7 KO cells, and the aberrant accumulation of p62 protein still existed after pretreatment with CHX (Fig. 5C, D), demonstrating that blocking degradation is the primary cause of p62 accumulation.

In addition, another transcriptional factor Nrf2 activated by p62 is taken as a pro-oncogenic protein [37, 38]. However, the RNA-seq data showed no difference in the target genes of Nrf2 between WT and ATG7 KO cells treated with H_2_O_2_ (Fig. 5E). The phosphorylation and nuclear translocation of Nrf2, and the mRNA level of main target genes (PRDX-1 and TXNDR-1) remained unchanged after autophagy was blocked (Figs. 5F and S9). Moreover, knockdown of Nrf2 expression had no significant effect on cell viability after exposure to H_2_O_2_ (Fig. 5G). These data ruled out the effect of Nrf2 on autophagy-facilitating apoptosis under oxidative stress.

### p62 initiates the activation of anti-apoptotic PKCι-IKK-NF-κB pathway in LECs

**Fig. 6:**
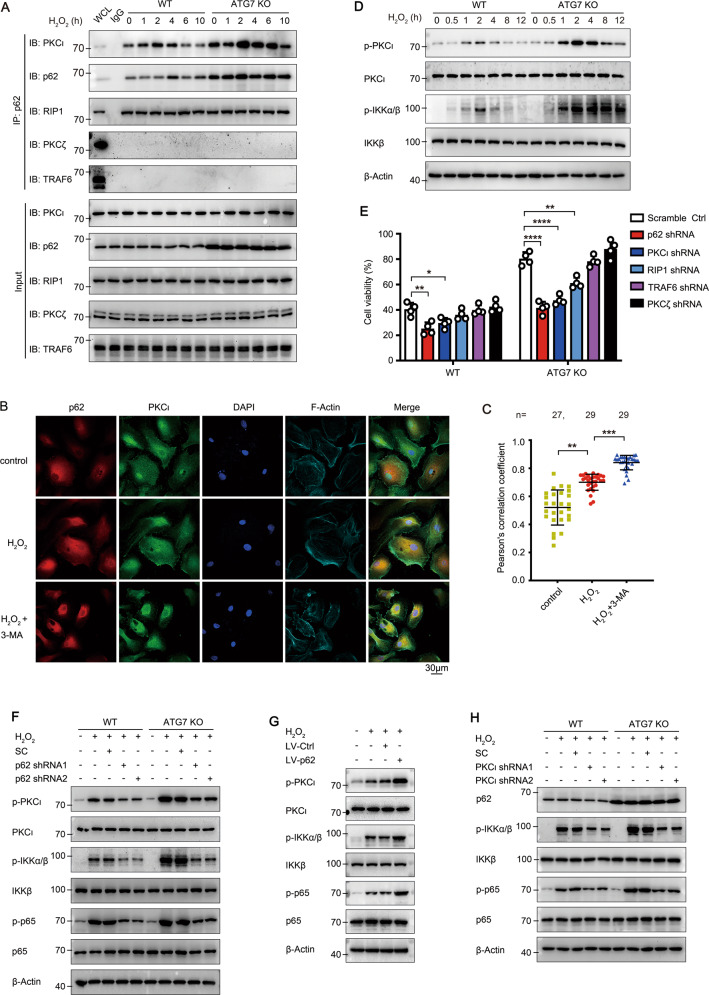
p62 serves as a scaffold protein to activate the PKCι-NF-κB pathway. **A** Endogenous interaction of p62 with PKCι and RIP1 in WT and ATG7 KO HLE-B3 cells treated with 200 μM H_2_O_2_ for indicated times. Cell lysates (Input; lower) and anti-p62 or anti-IgG immunoprecipitates (upper) were immunoblotted with the indicated antibodies. **B** Confocal images of p62 (red) and PKCι (green) co-localization in primary cultured human LECs from five elder healthy donors pretreated with 0.5 mM 3-MA for 3 h, followed by 50 μM H_2_O_2_ for another 1 h. LECs stained with DAPI (blue) and Phalloidin-iFluor 647 (F-Actin, cyan). Scale bar, 30 μm. **C** Co-localization of p62 and PKCι evaluated by Pearson’s correlation coefficient in primary cultured human LECs treated as in **B**. Mean ± SD; *n* = numbers of healthy donors analyzed; each symbol represents one cell; **p < 0.01, ***p < 0.001 (Kruskal-Wallis test followed by Bonferroni post hoc test). **D** Immunoblots showing total and phosphorylated PKCι and IKKα/β levels in WT and ATG7 KO HLE-B3 cells exposed to 200 μM H_2_O_2_ for indicated times. **E** Viability of WT and ATG7 KO HLE-B3 cells after knockdown of p62, PKCι, RIP1, TRAF6, or PKCζ expression exposed to 200 μM H_2_O_2_ for 24 h. Mean ± SD from four independent experiments, *p < 0.05, **p < 0.01, ****p < 0.0001 (one-way ANOVA followed by Bonferroni post hoc test). **F** Immunoblots showing total and phosphorylated PKCι, IKKα/β, and p65 levels in WT and ATG7 KO HLE-B3 cells before and after p62 RNAi, in the presence of 200 μM H_2_O_2_ for 2 h. **G** Immunoblots showing total and phosphorylated PKCι, IKKα/β, and p65 levels in WT and ATG7 KO HLE-B3 cells before and after p62 overexpression using lentiviral vector, in the presence of 200 μM H_2_O_2_ for 2 h. **H** Immunoblots showing total p62, IKKα/β, p65, and phosphorylated IKKα/β and p65 levels in WT and ATG7 KO HLE-B3 cells before and after PKCι RNAi, in the presence of 200 μM H_2_O_2_ for 2 h.

p62 specifically binds to atypical PKCs (aPKCs, including PKCι and PKCζ), and recruits RIP1 (receptor-interacting protein kinase 1) or TRAF6 (TNFα receptor-associated factor 6) to form an aPKC-p62-RIP1/TRAF6 complex to phosphorylate IKK. The interaction between endogenous p62 and PKCι detected by co-immunoprecipitation was significantly increased 2 h after H_2_O_2_ stimulation in WT cells, and was much stronger in ATG7 KO cells due to the over-accumulation of p62 (Fig. 6A). Besides, p62 also bound to RIP1, but not TRAF6 or PKCζ, in both KO and WT cells, indicating that the formation of p62-PKCι-RIP1 complex for NF-κB activation (Figs. 6A and S10A–C). More importantly, in primary cultured human LECs from healthy elderly donors, the co-localization level of p62 and PKCι was low under steady-state conditions, and markedly upregulated when treated with 50 μM H_2_O_2_, and was further elevated when pretreated with 3-MA (Fig. 6B, C). In addition, no significant difference in PKCι protein level in ATG KO and WT cells indicated no obvious autophagic degradation of PKCι (Fig. 6A). Collectively, these data demonstrated that oxidative stress promotes formation of the p62–PKCι complex, which is particularly prominent under conditions of deficiency or inhibition of autophagy.

We further confirmed whether p62–PKCι complex contributes to autophagy-facilitating apoptosis of LECs. Firstly, the phosphorylation of PKCι and IKKα/β was boosted after H_2_O_2_ stimulation and was further promoted after ATG7 or ATG3 ablation (Figs. 6D and S8A). In addition, knockdown of p65 did not affect the activation of upstream signal PKCι and IKKα/β (Fig. S7B). Secondly, knockdown of the scaffold protein p62 exacerbated H_2_O_2_-induced apoptosis and downregulated the phosphorylation of PKCι, IKKα/β, and p65 in both WT and ATG7 KO cells (Figs. S11A and 6E, F). Knockdown of p62 also promoted H_2_O_2_-induced cell senescence in WT cells (Fig. S11B). Meanwhile, p62 overexpression in WT cells enhanced the cell viability and activation of PKCι, IKKα/β and p65 (Figs. S12A, B and 6G). Thirdly, depletion of PKCι significantly decreased cell viability and IKK-NF-κB cascade activation in response to H_2_O_2_ in both WT and ATG7 KO cells (Figs. S12C and 6E, H). In addition, RIP1 depletion partially impaired the cell viability and IKK-NF-κB cascade activation (Figs. S12D–I and 6E). Together, these data demonstrated that p62 acts as the central signal hub to recruit PKCι and RIP1, and subsequently promotes the activation of PKCι-IKK-NF-κB cascade.

### Blockage of autophagy rescues age-related apoptosis of LECs through preventing p62 degradation

**Fig. 7:**
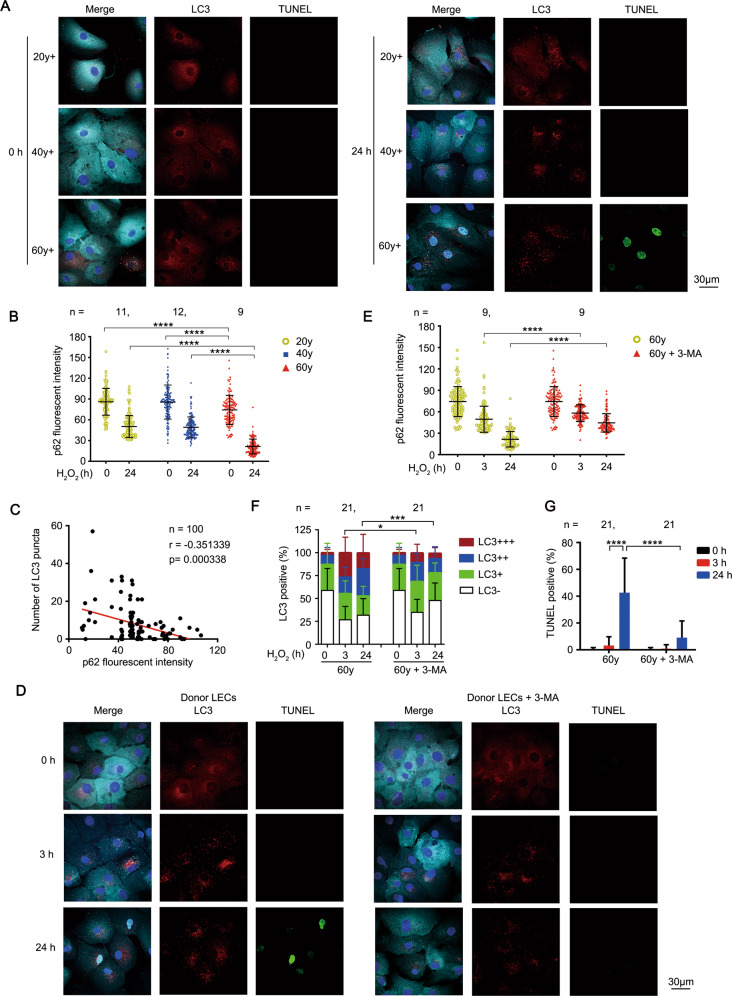
Blockage of autophagy rescues age-related apoptosis of LECs through preventing p62 degradation. **A** Representative confocal images of primary cultured human LECs from healthy donors of different ages stimulated with 50 μM H_2_O_2_ for indicated times. LECs are stained with LC3 antibody (red), p62 antibody(cyan), TUNEL labeling (green), and DAPI (blue). Scale bar, 30 μm. **B** Fluorescent intensity of p62 in primary cultured human LECs from healthy donors treated as in **A** (mean ± SD; *n* = numbers of healthy donors analyzed, ****p < 0.0001, Kruskal-Wallis test followed by Bonferroni post hoc test). **C** Correlation between p62 fluorescence intensity and the number of LC3 puncta in LECs from healthy donors of different age groups treated as in **A** (100 cells were analyzed; each symbol represents one LEC). *p* = 0.000338 by Spearman’s rank correlation test. **D** Representative confocal images of primary cultured human LECs from elder healthy donors pretreated with or without 0.5 mM 3-MA for 3 h, followed by 50 μM H_2_O_2_ exposure for indicated times. LECs are stained with LC3 antibody (red), p62 antibody(cyan), TUNEL labeling (green), and DAPI (blue). Scale bar, 30 μm. **E** Fluorescent intensity of p62 in primary cultured human LECs from elder healthy donors treated as in **D**. (mean ± SD; *n* = numbers of healthy donors analyzed, ****p < 0.0001, Mann-Whitney *U* Test). **F** Grouped stacked bars showing the percentages of LC3^-^, LC3^+^, LC3^+ +^, and LC3^+++^ in primary cultured human LECs from elder healthy donors treated as in **D** (mean ± SD; *n* = numbers of healthy donors analyzed; *p < 0.05, ***p < 0.001, Mann-Whitney *U* Test). **G** Percentages of TUNEL-positive primary cultured human LECs from healthy donors treated as in **D** (mean ± SD; *n* = numbers of healthy donors analyzed; ****p < 0.0001, two-way ANOVA followed by Bonferroni post hoc test).

There was nearly complete clearance of p62 concomitant with high autophagic activity and cell death in some LECs from senile cataract patients (Fig. 5A). In the dynamic model of primary cultured LECs, oxidative stress triggered more durable activation of autophagy in LECs from elder healthy donors than those from younger healthy donors as previously described (Fig. 2H). Accordingly, there was severe depletion of p62 protein in the elder LECs (60–70 years) at 24 h after H_2_O_2_ stimulation, while moderate depletion of p62 protein was observed in the young to middle-aged LECs (20–50 years) (Fig. 7A, B). The statistically significant negative correlation between p62 protein level and autophagy activity in LECs from all age groups were observed, confirming the strong regulation effect of autophagy on p62 protein level in LECs (Fig. 7C).

These findings allow us to speculate that durable autophagy excessively degrades p62 protein and impairs the PKCι-IKK-NF-κB pro-survival pathway, subsequently enhancing the sensitivity of LECs to oxidative stress-induced apoptosis. To evaluate the potential therapeutic effect, we further investigated whether blocking autophagy can rescue the H_2_O_2_-induced apoptosis in LECs from elderly healthy donors. Indeed, pretreatment with 3-MA, the inhibitor of early-stage autophagy, significantly down-regulated the autophagy level and simultaneously ameliorated the degradation of p62 protein at 3 h and 24 h after H_2_O_2_ stimulation (Fig. 7D–F). Hence, the apoptosis of elder LECs was effectively inhibited at 24 h after H_2_O_2_ stimulation (Fig. 7G). These data suggested autophagy was a potential target for controlling apoptosis in senescent LECs.

## Discussion

Senile cataract, a typical senescence-associated disease in all elder individuals, is an ideal model to investigate the molecular mechanisms for senescence and age-related cell death. LECs, the only type cell in the lens, are critical to senile cataract formation [10]. Meanwhile, the lens capsule tissue containing LECs has good accessibility for relevant research, as it can be obtained during routine cataract surgery. Importantly, since the lens is an immunologically privileged site, the factor of “inflammaging” induced by immune cells, which is recently thought to be linked to a myriad of age-related diseases, can be ruled out in the ageing process of LECs [1].

Our work suggested that, in senescent LECs, prolonged autophagy in response to oxidative stress led to apoptotic cell death via excessive degradation of p62 and inhibition of the pro-survival PKCι-IKK-NF-κB signaling, hence contributed to the senile cataract formation. These findings established the link among autophagy, apoptosis and senile cataract formation, and highlighted the importance of autophagy-facilitating cell death in the pathogenesis of age-related diseases.

The potential relationship between autophagy and ageing is controversial. For LEC senescence, our data demonstrated that Atg7 knockout significantly suppressed H_2_O_2_-induced senescence, and this inhibitory effect was almost fully abrogated by knockdown of p62 (Figs. 2B and S11B), suggesting a p62-dependent pattern. For age-related cell death, we propose a novel mechanism that autophagy promoted age-related cell death via degrading p62 and suppressing the pro-survival p62-PKCι-NF-κB pathway. To validate the mechanism, two diverse human primary LECs models were used. One is the fixed capsule model from senile cataract patients, in which different autophagy levels were detected among the LECs from the same patient, and higher autophagy was found to be associated with lower p62 protein level and cell death. The other is the dynamic model of the primary cultured LECs spanning from younger to elder stage. In this model, oxidative stress was found to induce more durable activation of autophagy, more obvious p62 degradation and higher percentage of dead cell in the elder LECs than those in the younger LECs.

Of note, more durable autophagy was detected in the elder LECs than in the young to middle-aged LECs, although the autophagic level was not significantly different between them. These data indicated that elevation of autophagy is an early response to the impaired intracellular homeostasis induced by oxidative stress, and subsequently, the younger LECs tend to reach a new homeostatic balance with a decreased level of autophagy, while the older LECs maintain a high, detrimental level of autophagy that induces apoptosis. However, the mechanism for more durable autophagy in the elder LECs is unclear. Referring to recent literatures, some robust theories like “garbage accumulation” may explain our findings. The “garbage”, including dysfunctional organelles and protein aggregates, can accumulate in senescent cells and progressively impair cellular homeostasis [39]. Compared with younger cells, senescent cells exhibit decreased threshold of autophagy due to the higher basal level of “garbage”, or undergo the prolonged activation of autophagy due to the requirement to eliminate more “garbage” [40–42]. Further studies are desirable to elucidate the molecular mechanisms underlying the hyperactivation of autophagy in senescent cells.

**Fig. 8:**
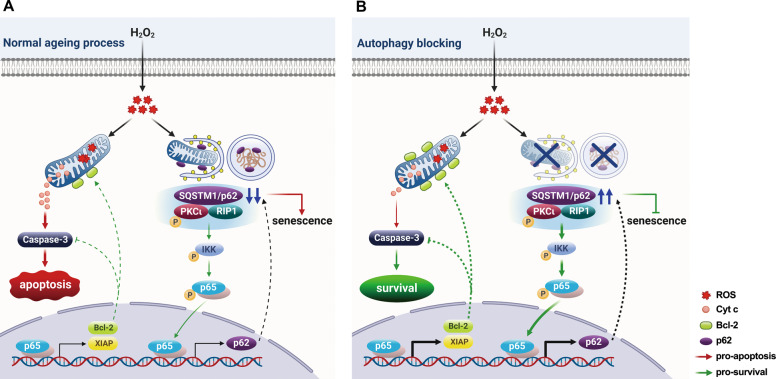
Working model illustrating the role of autophagy in oxidative stress-induced senescence and cell death. **A** Oxidative stress triggers durable autophagy in human LECs which leads to excessive degradation of p62 protein, and subsequently results in premature senescence. Low level of p62 scaffold protein can not sustain the pro-survival PKCι-IKK-NF-κB signaling, and hence facilitates apoptosis induced by oxidative stress. **B** Autophagy is inhibited by genetic deletion of the Atg gene or a chemical inhibitor. The elevated level of p62 delays the senescence process, and antagonizes pro-apoptotic signaling via boosting the sufficient pro-survival signaling PKCι-IKK-NF-κB cascades.

In summary, our work described a model illustrating the effect of autophagy on age-related LECs death in senile cataract (Fig. 8). During ageing process of human LECs, oxidative stress triggers durable activation of autophagy and leads to excessive degradation of p62 protein, subsequently resulting in premature senescence. As a scaffold protein, low p62 level cannot sustain the sufficient pro-survival signaling PKCι-IKK-NF-κB cascades, causing aggravation of apoptosis induced by oxidative stress (Fig. 8A). With autophagy being blocked, accumulated p62 protein delays the senescence process, and antagonizes the pro-apoptotic signaling via boosting the sufficient pro-survival signaling PKCι-IKK-NF-κB (Fig. 8B). Our effort to elucidate the role of autophagy in LECs senescent process and cell death are contributory to the better understanding of etiology underlying senile cataract as well as other age-related diseases.

## Materials and methods

### Isolation of human and mouse lens capsules

The human lens capsule epithelium specimens were obtained from the Department of Ophthalmology, the Affiliated Hospital of Zhejiang University, from February 2019 to September 2020. The specimens were categorized into three groups according to the source: (1) Senile cataract group: lens capsule epithelium specimens of senile cataract were from patients receiving cataract surgery, who were further divided into four subgroups—mild, moderate A, moderate B, and severe—according to the grade of cataract determined by the Lens Opacities Classification System III (LOCS III standard) [43] (Table S1). (2) Transparent lens group: specimens of transparent lens from patients without cataract, who received transparent lens extraction surgery for the purpose of presbyopia correction, serving as the negative controls for the senile cataract patient group. (3) Healthy donor group: specimens of transparent lens from the healthy donor eyes, which were prepared for corneal transplantation surgery within 2 h after decease, and were used for primary cell culture; these specimens were further divided into three subgroups—young, middle-aged and old (Table S1). Exclusion criteria for all participants were: (i) congenital, traumatic, and metabolic-type cataracts; (ii) any history of previous ocular surgery; and (iii) concomitant systemic disorders like diabetes mellitus and rheumatologic disease. The maneuver of capsulorhexis to obtain lens capsule specimens during surgery was performed by the same experienced cataract specialist (WH).

For immunofluorescence staining, the capsule specimens were promptly fixed in 4% paraformaldehyde (PFA) within 10 s after capsulorhexis during cataract phacoemulsification surgery. For primary LEC culture, human lens capsule epithelium specimens were washed three times in PBS and immediately placed in Eppendorf tubes containing standard culture medium (Advanced DMEM/F12, Gibco, NY, USA) supplemented with 10% fetal bovine serum (FBS) (Cat.# 10099-141, Gibco), 2 mM GlutaMAX^TM^ (Cat.# 35050-061, Gibco), and 100 U/ml penicillin and streptomycin (Cat.# 15140-122, Gibco). These specimens were transferred to the laboratory within 1 h for further cultivation. GFP-LC3 transgenic C57BL/6 mice were anesthetized by intraperitoneal injection of 80 mg/kg pentobarbital sodium and sacrificed by dislocating the cervical vertebra. The lenses were isolated from 8-week-old female mice and capsulorhexis was performed.

The human and animal studies were approved by the Research Ethics Committee of the Affiliated Hospital, School of Medicine, Zhejiang University (No. 2019-267 and No. 2019-23-1). The human studies were conducted in accordance with the Declaration of Helsinki. Signed informed consents were obtained from all participants.

### Primary LEC and cell line cultures

Lens capsule epithelium specimens were cut into 2 mm × 2 mm pieces, placed on 24-well culture plates (Corning, NK, USA), and incubated in a humidified atmosphere of 5% CO_2_ at 37 °C. Movement of the plates was avoided during the first week of culture to allow the capsules to attach to the bottom of culture wells. Human LECs that migrated ~5 mm from the capsular edge one week later were prepared for further experiments.

HLE-B3 cells were cultured in DMEM/F12 supplemented with 10% FBS. HEK 293T cells (purchased from Cell Bank of Chinese Academy of Sciences) were maintained in DMEM supplemented with 10% FBS.

### Senescence-associated β-galactosidase assay

The assay was performed using the SA-β-gal Staining Kit (Cat.# C0602, Beyotime, Shanghai, China). In brief, the cells were washed, fixedand incubated overnight at 37 °C with staining solution containing X-gal. Then the staining was imaged and quantified under a regular light microscope.

### Generation of CRISPR/Cas9 knockout cell lines

sgRNA sequences for the generation of CRISPR/Cas9-mediated knockout cell lines were designed using CRISPR design software (http://crispr.mit.edu). sgRNA sequences targeting the human ATG7 gene (5′-GAAGTTGAACGAGTACCGCC-3′) or the human ATG3 gene (5′-AATGTGATCAACACGGTGAA-3′) were cloned into a pEP-KO Z1779 vector. HLE-B3 cells were transfected with pEP-ATG7-KO or pEP-ATG3-KO vector using FuGENE HD Transfenction Reagent (Cat.# E2311/2, Promega, WI, USA), according to the manufacturer’s protocol. Two days post-transfection, cells were cultured in complete culture medium containing 800 ng/ml puromycin (Cat.# P7255, Sigma, Germany) for 7 days. Individual clones were picked and analyzed by western blotting to confirm deletion of the ATG7/3 genes.

### Lentiviral transduction

The lentivirus transfer plasmid pLVX-shRNA (shRNA sequences shown in Table S2) was transfected into HEK 293T cells with packaging plasmids pLP-VSVG, pLP1, and pLP2 using PolyJet Reagent (Cat.# SL100688, SignaGen, USA) to generate lentiviral particles. Forty-eight hours later, the lentivirus-containing supernatants were collected, filtered through a 0.45-μm filter, and concentrated by ultracentrifugation. Subsequently, 1 × 10^5^ HLE-B3 cells were seeded in each well of six-well plates and the lentiviral supernatant and 3 μg/ml polybrene (Cat.# HB-PB-1000, HANBIO, Shanghai, China) were added. Eight hours after transduction, the lentiviral supernatant was removed and replaced by fresh complete culture medium. Two to three days after transduction, the efficiency of RNA interference was determined by Western blot and the cells were used for further experiments.

The lentivirus expressing human p62 protein was packaged by GeneCopoeia Co. (LPP-M0245-Lv105-100). The overexpression of p62 was confirmed by Western blotting.

### Immunofluorescence microscopy

The lens capsule specimens from the cataract group and transparent lens control group were immediately fixed with 4% PFA for 15 min at room temperature (RT), and then washed three times with PBS. The specimens were permeabilized with 0.1% saponin (Cat.# 47036, Sigma) in PBS for 20 min and blocked with 10% bovine serum albumin in PBS for 1 h at RT. The specimens were incubated with appropriate primary antibodies (Table S3) for 16 h at 4 °C, washed three times, and then incubated with secondary antibodies for 1 h at RT. The specimens were rinsed again and stained using a TUNEL Apoptosis Detection Kit (Cat.# 40306ES60, Yeasen Biotech, Shanghai, China) according to the manufacturer’s instructions. The lens capsule specimens were flattened on glass coverslips and mounted on slides with ProLong™ Diamond Antifade Mountant with DAPI (Cat.# P36966, Invitrogen, WA, USA).

Primary cultured human LECs or mouse LECs were seeded on coverslips placed in 24-well plates. After 50 μM H_2_O_2_ treatment for the indicated time, the LECs were fixed, permeabilized, and blocked. After washing, the LECs were sequentially incubated with appropriate primary antibodies and secondary antibodies, and subsequently stained with Phalloidin-iFluor 647 reagent (Cat.# ab176759, Abcam, Cambridge, UK) and/or TUNEL.

Fluorescence signals were acquired with an Olympus high-resolution laser scanning confocal microscope (IX83-FV3000-OSR) or a Nikon resonant scanning confocal microscope (A1R). To compare the fluorescence intensity between different samples, the laser intensity and pinhole were fixed for all samples. To quantify LC3 puncta, four randomly-selected images from each sample were analyzed using ImageJ software (1.52p). LC3 puncta were defined if the intense fluorescence area was >0.1 µm^2^. The cultured LECs were categorized according to the grade of LC3 staining in Figs. 2 and 7: (1) LC3^-^, no puncta in the cytoplasm; (2) LC3^+^, LC3 aggregates around nuclei but <5 puncta; (3) LC3^++^, 5 to 15 puncta; and (4) LC3^+++^, >15 puncta. At least 100 cells from each group were enumerated. Co-localization was analyzed using Huygens Professional software (version 20.04, Scientific Volume Imaging).

### Transmission electron microscopy (TEM)

The lens capsule specimens from cataract patients were immediately fixed in 2.5% glutaraldehyde in 0.1 M phosphate buffer (Cat.# G5882, Sigma) for 2 h at RT. After thorough rinsing in 0.1 M phosphoric acid solution, the specimens were post-fixed with 1% osmium tetroxide (Cat.# 365092, Sigma) for 1 h and rinsed three times in water. The specimens were then stained with 2% uranyl acetate for 0.5 h at 4 °C before dehydration through a graded ethanol series. Embedding agent and pure acetone (1:1, 3:1) were added for gradient embedding, and specimens were finally mixed with pure embedding agent and incubated at 37 °C. The specimens were cut using an ultra-thin trimming machine and the ultrastructural features of autophagy were observed and imaged under a Tecnai transmission electron microscope (FEI, USA) at an operating voltage of 80 kV.

### Cell viability assay

HLE-B3 cells were seeded at 6000 cells per well in 96-well plates and then exposed to different doses of H_2_O_2_ or UV radiation for the desired periods of time. All experiments were performed in triplicate and repeated for at least three times. Cell viability was quantified using a CCK8 Assay Kit (Cat.# CK04, Dojindo, Japan) according to the manufacturer’s instructions: absorbance was read at 450 nm using a Model 680 Microplate Reader (Bio-Rad, CA, USA).

### RNA sequencing analysis

Total RNA was extracted from HLE-B3 cells with or without exposure to 200 μM H_2_O_2_ for 12 h. Sequencing libraries were generated using NEBNext® UltraTM RNA Library Prep Kit for Illumina® (NEB, USA) and library quality was assessed on the Agilent Bioanalyzer 2100 system. Gene expression was normalized to Fragments Per Kilobase Million and differential expression analysis of two groups (three biological replicates per group) was performed using the DESeq2 R package (1.16.1). Reliably-expressed genes (Fragments Per Kilobase Million >1) with an adjusted *p* value < 0.01 and |log_2_FC| > 1 found by DESeq2 were assigned as differentially-expressed genes. KEGG pathway enrichment analyses of differentially-expressed genes were performed by DAVID (https://david.ncifcrf.gov/) [44]. The cutoff was set at a *p* value <0.05. Genes in ATG7 KO and WT groups (H_2_O_2_ treated and untreated) in significant pathways were normalized to produce hierarchical clustering and generate heatmaps. All original data were deposited in the NCBI’s Gene Expression Omnibus database (GSE161701).

### RNA isolation and real-time PCR

Total RNA was extracted from HLE-B3 cells using TRIzol reagent (Invitrogen, 10296010), and quantified with NanoDrop2000 (Thermo). Reverse transcription was performed with a PrimeScript^TM^ RT reagent Kit with gDNA eraser (Cat.# RR047A, Takara, Japan) according to the manufacturer’s instructions. Real-time quantitative PCR was performed using an iTaq Universal SYBR Green RT-PCR Kit (Cat.# 1725122, Bio-Rad) and a CFX96 Real-Time PCR Detection System (Bio-Rad). The primer sequences are listed in Table S4. All samples were run in triplicate and data were normalized by the β-actin mRNA level. The experiments were repeated at least three times.

### Co-immunoprecipitation and Western blot

HLE-B3 cells were cultured in 10 cm plates (3 × 10^6^ cells) for 24 h and exposed to 200 μM H_2_O_2_ for the desired period of time. Cells were collected and lysed in ice-cold NP-40 buffer (Cat.# P0013F, Beyotime) containing 1 mM PMSF (Cat.# ST506, Beyotime) and Phosphatase Inhibitor Cocktail (Roche, 4906837001). The lysates were reacted with 2 μg/ml of different antibodies (anti-p62, PKCι, RIP1, or TRAF6 antibodies, Table S3) and incubated overnight at 4 °C. Then protein G dynabeads (Cat.# 78609, Thermo) were added, incubated for another 2 h at RT, and washed at least three times with IP buffer. Immuno-precipitated samples were run on SDS-PAGE gel and immunoblotted.

For Western blot, cell lysates were prepared using RIPA buffer (Cat.# 9806, Cell Signaling Technology, MA, USA) containing PMSF and Phosphatase Inhibitor Cocktail. Protein concentrations were determined using a BCA Protein Assay Kit (Cat.# CW0014S, Cwbio, Beijing, China). The protein samples were separated by 8–12% SDS-PAGE gel and then electrotransferred to PVDF membranes (Cat.# IPVH00010, Merck Millipore, Germany) for 1.5 h at 0.3 A current. The membranes were blocked in 5% skimmed milk in TBST (0.05% Tween-20 in Tris-buffered saline) for 1 h at RT, and then incubated with the indicated antibodies (Table S3) for 16 h at 4 °C. After incubation with HRP-conjugated secondary antibodies, the membranes were assessed with the Alpha chemiluminescence gel imaging system (FluorChem E, Cell Biosciences, USA). β-actin was used as the protein loading control.

### Nuclear and cytoplasmic protein extraction

Nuclear and cytoplasmic protein extraction was performed at 4 °C using a Nuclear and Cytoplasmic Extraction Kit (Cat.# C500009, Sangon Biotech, Shanghai, China) according to the manufacturer’s instructions. Briefly, HLE-B3 cells were collected and re-suspended in 200 μl hypotonic buffer containing 1 mM DTT, 1 mM PMSF, and Phosphatase Inhibitor Cocktail for 20 min incubation. After centrifugation at 800 g for 10 min, the supernatant containing cytoplasmic protein was carefully transferred to a new tube. The mucoid sediment containing nuclei was washed twice in hypotonic buffer, and re-suspended in 25 μl lysis buffer containing 1 mM DTT, 1 mM PMSF, and Phosphatase Inhibitor Cocktail for 20 min incubation. The supernatant containing nuclear protein was collected after centrifugation at 12000 g for 10 min.

### Flow cytometry

Apoptosis assays of HLE-B3 cells were performed using the Annexin V-FITC/PI apoptosis Kit (Cat.# 70-AP101-100, Multi Sciences, Zhejiang, China) according to the manufacturer’s protocol. Numbers of Annexin V and PI-positive cells were detected by ACEA Novocyte flow cytometry (ACEA Biosciences, CA, USA) and data were processed with FlowJo software (v10.4.0).

For intracellular ROS detection, 2 × 10^5^ cells were exposed to 200 μM H_2_O_2_ for the indicated time in the presence or absence of 10 mM NAC (Cat.# A9165, Sigma). The cells were then incubated with 10 mM 2′,7′-dichlorodihydrofluorescein diacetate (Cat.# D6883, Sigma) in darkness for 30 min and fixed in 4% PFA at RT. Samples were analyzed for green fluorescence (DCFH) using flow cytometry.

For mitochondrial mass assessment, 2 × 10^5^ cells were exposed to 200 μM H_2_O_2_ for the indicated time and then incubated with 200 nM MitoTracker Green probe (Cat.# C1048, Beyotime,) for 30 min at 37 °C. The cells were collected and washed twice in PBS. The green fluorescence was measured by flow cytometry.

For cell-cycle analysis, 7 × 10^5^ cells were exposed to 200 μM H_2_O_2_ for the indicated time. The cells were collected and washed twice with ice-cold PBS, and then fixed in 70% ethanol overnight at 4 °C. The precipitate was collected and intensively washed twice with ice-cold PBS. After incubation with 50 μg/ml RNase I (Cat.# R4875, Sigma) at 37 °C for 30 min, the cells were stained with 100 μg/ml PI (Cat.# P4170, Sigma) in darkness for 15 min at RT. The percentage of cells in each phase of the cycle was determined by flow cytometry.

### Measurement of MDA, SOD, and GSH

After exposure to 200 μM H_2_O_2_ for the indicated time, cells were collected and sonicated in ice-cold PBS (pH 6.8) containing 1 mM PMSF to obtain cell homogenates. The supernatants of homogenates were prepared for MDA, SOD, and GSH assessment using assay kits (Jiancheng Biochemical Inc., Jiangsu, China). The MDA level was measured by detecting the thiobarbituric acid reacting substances at a wavelength of 530 nm (MDA Assay Kit, A003-1). SOD activity was detected using the xanthine oxidase method and determined by the absorbance at 450 nm wavelength (SOD Assay Kit, A001-3). After the reduced glutathione in the homogenate reacted with disulfide dinitrobenzoic acid and yielded a yellow compound, the intracellular level of GSH was measured as the absorbance at 405 nm (GSH Assay Kit, A006-2-1). All values were normalized to the total protein concentration of the same sample.

### Statistics

Statistical analysis was performed by SPSS software (version 26.0, IBM Corp.). Data were presented as mean ± SD. Each dataset was examined for normal distribution by Kolmogorov–Smirnov normality test. If data were normally distributed, student’s unpaired *t* test was performed for comparisons between two groups. For comparisons among more than two groups, one-way ANOVA was performed, followed by Bonferroni post hoc test. Two-way ANOVA followed by Bonferroni post hoc test was conducted when there are more than two independent variables. If data were non-normally distributed, Mann-Whitney *U* test was conducted for comparisons between two groups. Kruskal–Wallis test followed by Bonferroni post hoc test was conducted for comparisons among more than two groups. For comparisons of categorical variables, Pearson’s chi-square (χ^2^) test was performed. Correlation between p62 and LC3 flourescent intensity was tested with Pearson’s correlation analysis. Correlation between p62 flourescent intensity and number of LC3 puncta was tested with Spearman’s rank correlation analysis. All the statistical tests were two-sided. A *p* value of less than 0.05 was considered statistically significant. All experiments were carried out at least three times unless otherwise stated. The statistical details (such as statistical tests used) and repeated times of the experiments are given in the figure legends.

## Supplementary information

Supplementary Information

Reproducibility checklist

Declaration of contributions

## Data Availability

All data generated or analyzed in this study were included in this published article (and its supplementary files). The RNA-seq data had been deposited in the Gene Expression Omnibus database [GEO:GSE161701].
