# Supplementary material for: Autophagy facilitates age-related cell apoptosis—a new insight from senile cataract
Source: Cell Death Dis. 2022 Jan 10;13(1):37. doi: 10.1038/s41419-021-04489-8 (PMC8748728; doi:10.1038/s41419-021-04489-8)
Supplement: Supplementary file 1 — Supplementary Information [file 41419_2021_4489_MOESM1_ESM.docx]

Supplementary information for

**Autophagy facilitates age-related cell apoptosis - a new insight from senile cataract**

Jiani Huang, Wangshu Yu, Qin He, Xiaoying He, Ming Yang, Wei Chen, Wei Han

**This file includes：**

Supplementary Figure S1 to S12

Supplementary Table S1 to S4**
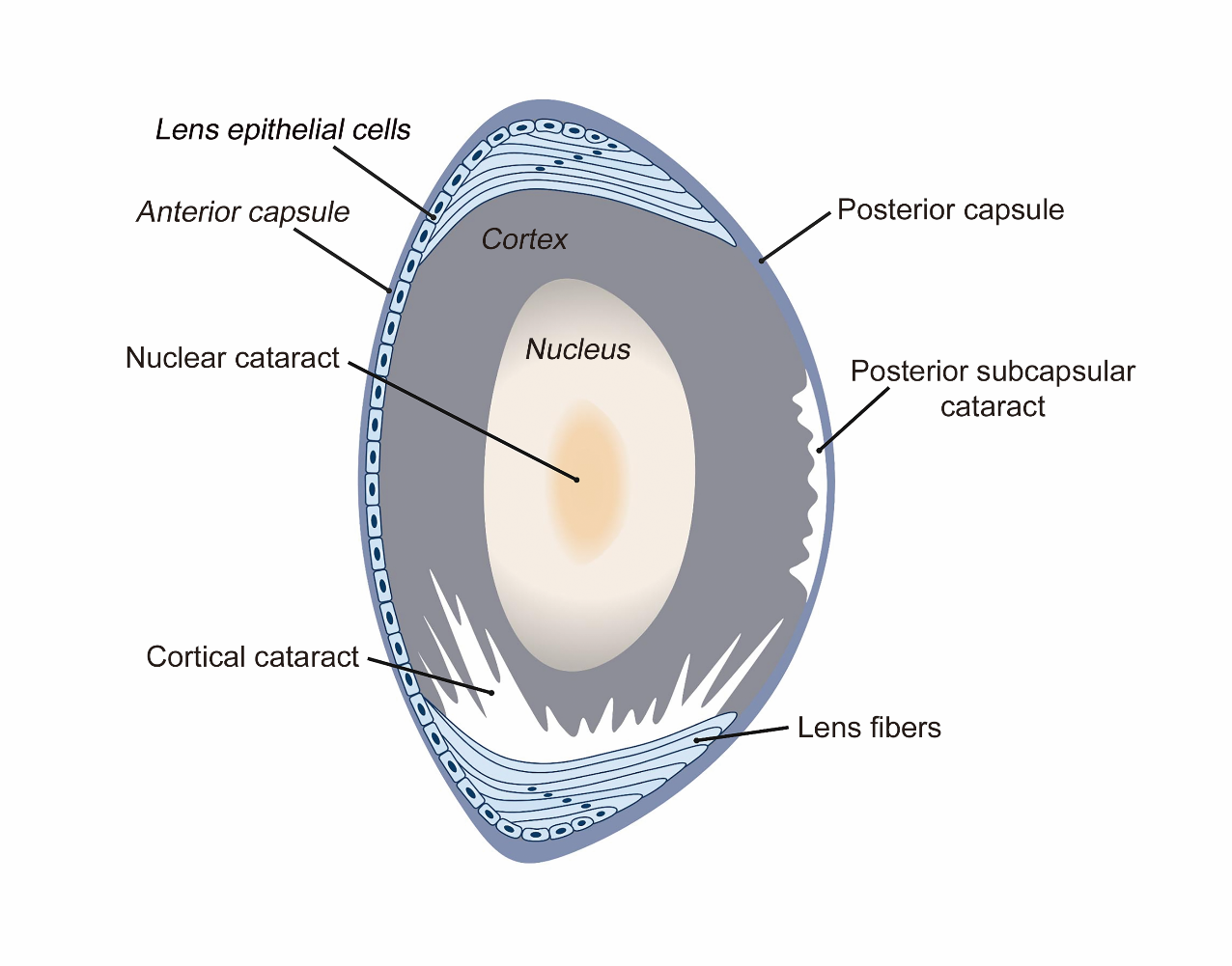
Figure S1. Diagram of the anatomical structure of the lens.** The mature lens is composed of: (1) the lens capsule, an elastic membrane surrounding the lens; (2) one layer of lens epithelial cells (LECs) lining the anterior-inner surface of the lens capsule; and (3) the lens cortex and nucleus which consist of non-organelle lens fibers. The LECs in the central zone of anterior capsule migrate to the peripheral zone where they gradually differentiate into organelle-free fibers towards the central core of lens. Cataract is classified into cortical, nuclear, and posterior subcapsular types according to the site of opacification in the lens. The figure was created with Biorender.

**
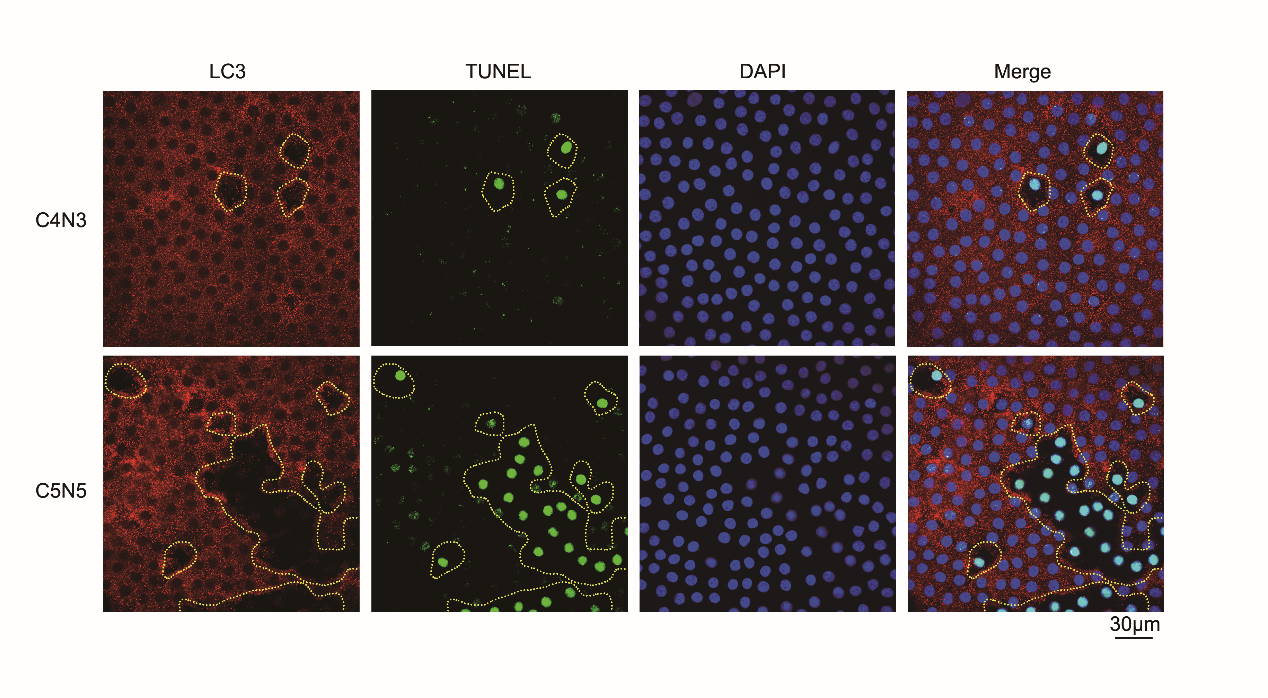
Figure S2. Patches of apoptotic cells in lens capsules from senile cataract patients.** Representative confocal images of LECs from 67 patients with moderate to severe grade senile cataract stained with LC3B antibody (autophagosome, red), TUNEL labeling (DNA fragment, green), and DAPI (nucleus, blue). Yellow dotted irregular circles indicate LC3^-^ TUNEL^++^ cells. Scale bar, 30 μm.

**
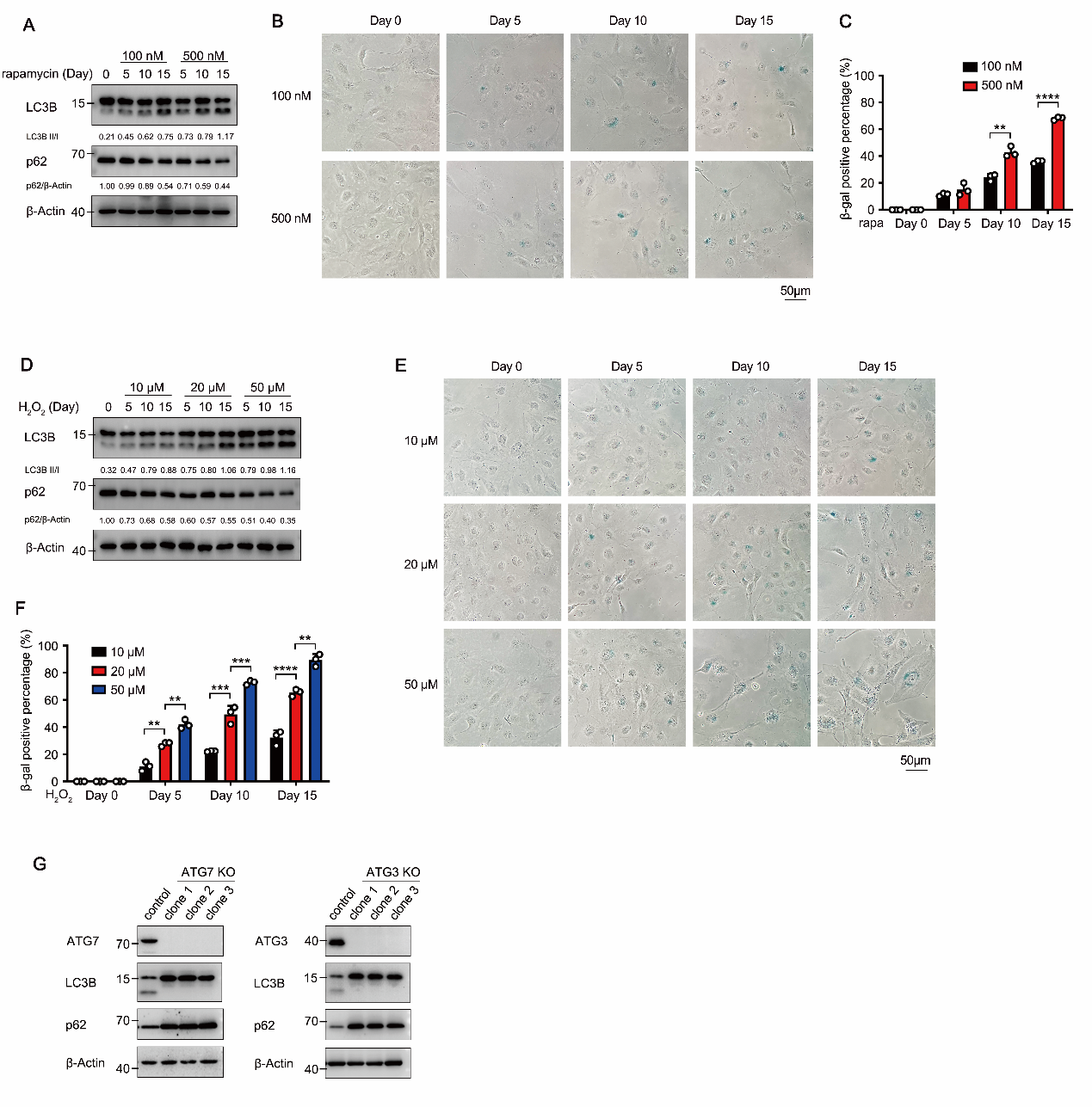
Figure S3. Autophagy promotes the cellular senescence of LECs. (A)** Immunoblots showing LC3B and p62 levels in HLE-B3 cells stimulated with different concentrations of rapamycin for indicated days. **(B)** β-galactosidase (β-gal) staining of HLE-B3 cells at indicated days post different doses rapamycin treatment was imaged by microscopy. Scale bar, 50 μm. **(C)** Quantification of the percentages of cells with β-gal positive staining treated as in (B). Data are mean ± SD from three random fields; ***p <0.01, ****p <0.0001* (unpaired Student’s t-test). **(D)** Immunoblots showing LC3B and p62 levels in HLE-B3 cells stimulated with different concentrations of H_2_O_2_ for indicated days. **(E)** β-gal staining of HLE-B3 cells at indicated days post different doses H_2_O_2_ treatment was imaged by microscopy. Scale bar, 50 μm. **(F)** Quantification of the percentages of cells with β-gal positive staining treated as in (E). ***p <0.01, ***p <0.001, ****p <0.0001* (one-way ANOVA followed by Bonferroni post hoc test). **(G)** Immunoblots showing CRISPR/Cas9-mediated knockout of ATG7 (left) and ATG3 (right) in HLE-B3 cells as well as LC3B and p62 levels. Three different knockout clones were used.

**
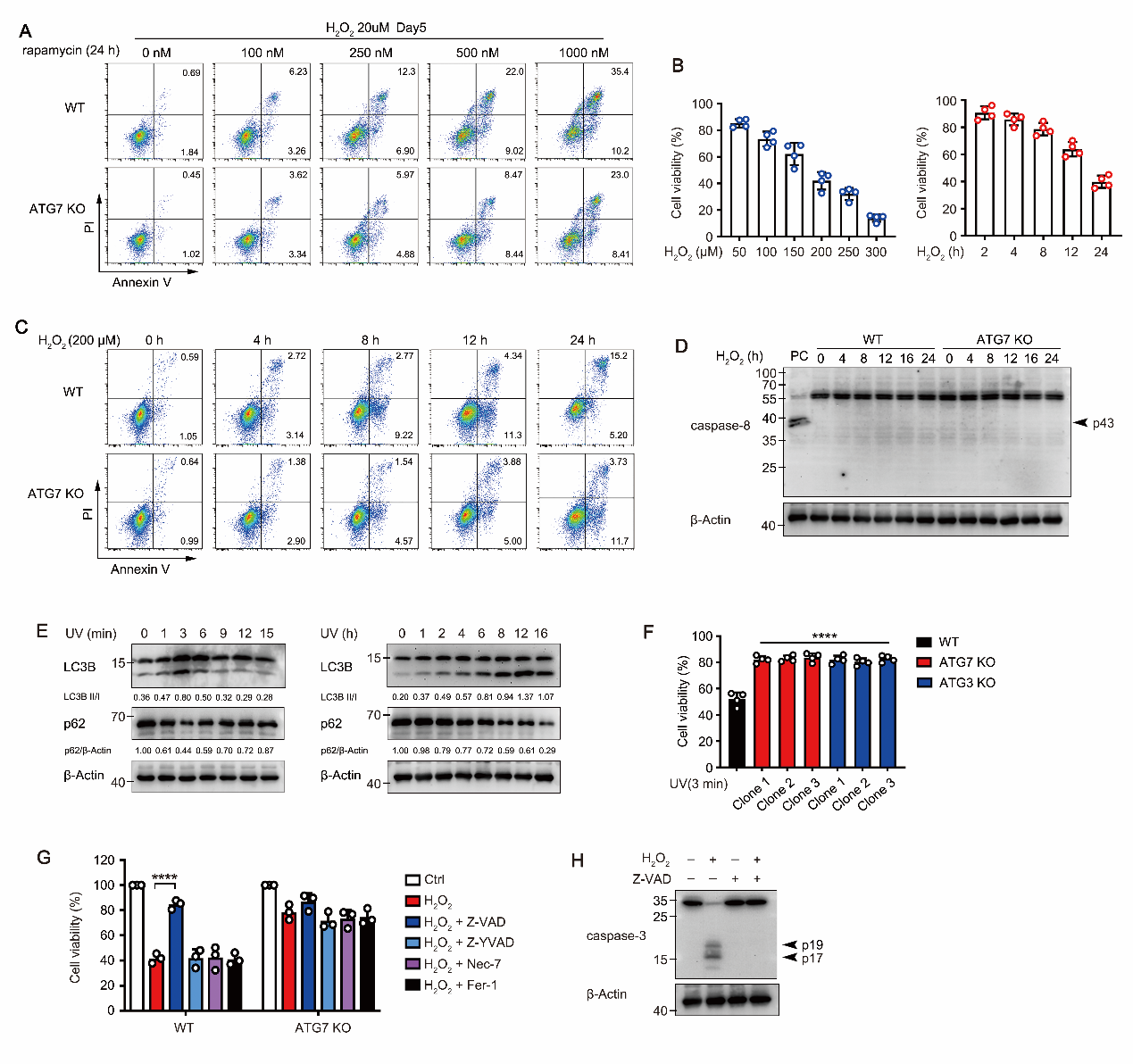
Figure S4. Oxidative stress induced apoptosis and increased autophagy level in HLE-B3 cells. (A)** Annexin V/PI staining for apoptosis in WT (upper) and ATG7 KO (lower) HLE-B3 cells pretreated with 20 μM H_2_O_2_ for 5 days, followed by rapamycin treatment in indicated concentrations for another 24 h. **(B)** Viability of HLE-B3 cells stimulated with different concentrations of H_2_O_2_ for 24 h (left) or with 200 μM H_2_O_2_ for indicated times (right) determined by CCK8 assay. **(C)** Annexin V/PI staining for apoptosis in WT (upper) and ATG7 KO (lower) HLE-B3 cells treated with 200 μM H_2_O_2_ for indicated times. **(D)** Immunoblots of activated caspase-8 (arrowhead) in WT and ATG7 KO HLE-B3 cells exposed to 200 μM H_2_O_2_ for indicated times. Jurket cells treated with 1 μM staurosporine for 3 h as the positive control (PC) of caspase-8 cleavage. **(E)** Immunoblots showing LC3B and p62 levels in HLE-B3 cells cultured for 12 h after receiving different doses of UV irradiation (left) or cultured for indicated times after receiving 3 min UV irradiation (right). **(F)** Viability of WT, ATG7 KO and ATG3 KO HLE-B3 cells cultured for 24 h after receiving 3 min UV irradiation determined by CCK8 assay. Mean ± SD from four independent experiments; ****p <0.0001 (one-way ANOVA followed by Bonferroni post hoc test). **(G)** Viability of WT and ATG7 KO HLE-B3 cells pretreated for 2 h with 20 μM Z-VAD, 10 μM Z-YVAD, 1 μM necrostatin-7 (Nec-7), or 1 μM ferrostatin-1 (Fer-1), followed by 200 μM H_2_O_2_ exposure for 24 h. Mean ± SD from three independent experiments; ****p <0.0001 (one-way ANOVA followed by Bonferroni post hoc test) **(H)** Immunoblots of caspase-3 in HLE-B3 cells pretreated with 20 μM Z-VAD for 2 h, followed by 200 μM H_2_O_2_ exposure for 16 h.

**
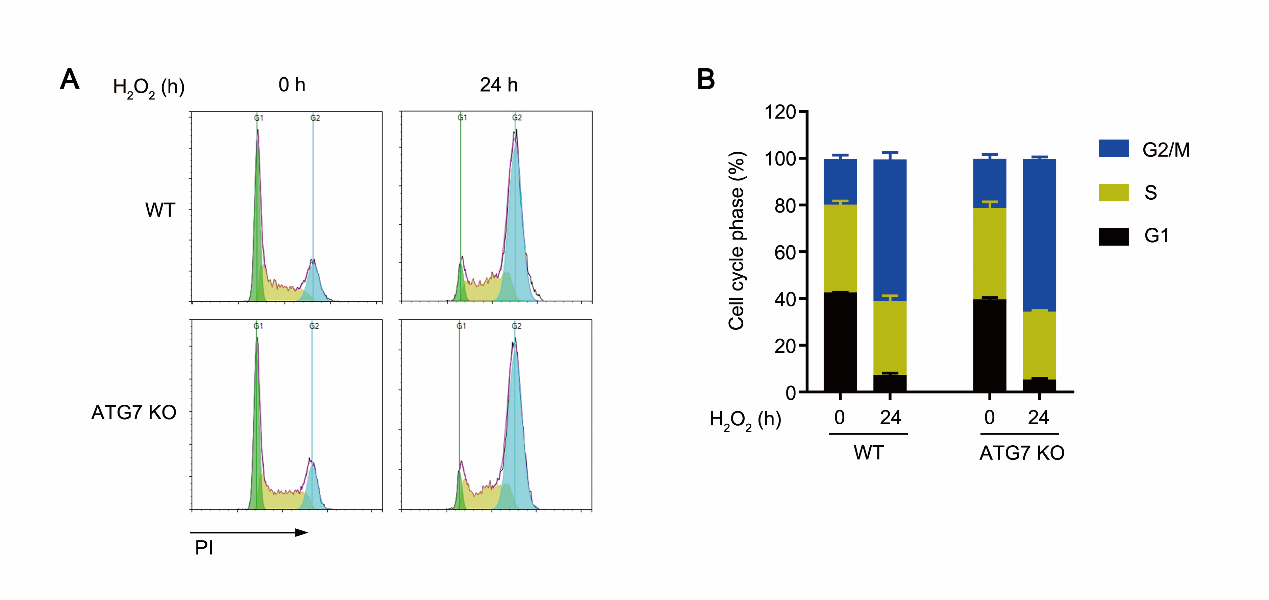
Figure S5. Ablation of ATG7 not alter the cell-cycle in HLE-B3 cells challenged by oxidative stress. (A)** Representative cell-cycle distribution in WT and ATG7 KO HLE-B3 cells with or without 200 μM H_2_O_2_ exposure determined by PI labeling and FACS analysis. **(B)** Grouped stacked bars showing the percentages of G1, S, and G2/M phases from three independent experiments (mean ± SD, *p* >0.05, χ^2^ test).

**
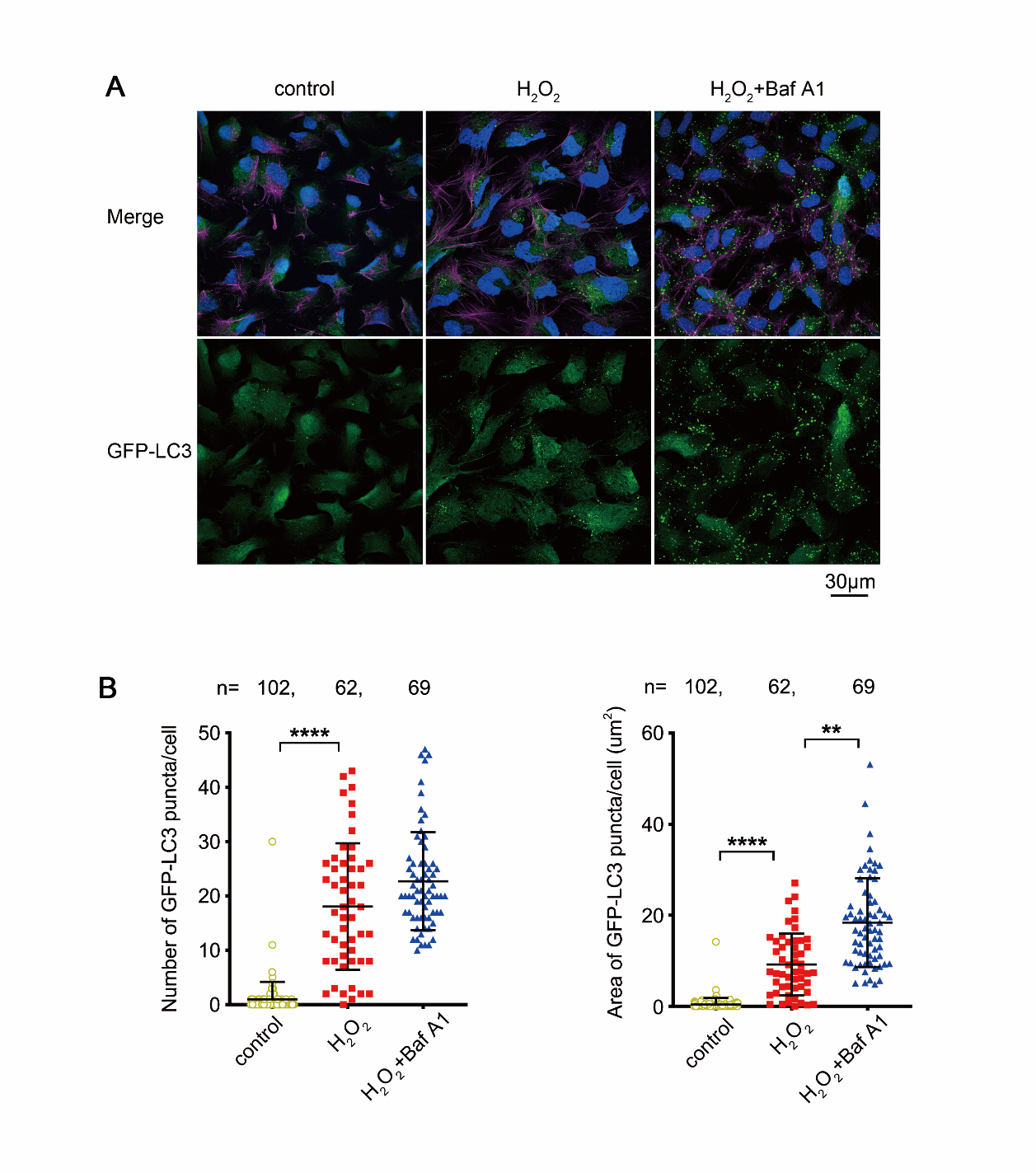
Figure S6. Autophagic flux induced by oxidative stress in primary cultured mouse LECs. (A)** Representative confocal images of primary LECs from GFP-LC3 transgenic mice pretreated with 100 nM Baf A1 for 4 h, followed by 50 μM H_2_O_2_ treatment for another 3 h. LECs are stained with DAPI (nucleus, blue) and Phalloidin-iFluor 647 (F-Actin, magenta). Scale bar, 30 μm. **(B)** Numbers of LC3 puncta (area of intense fluorescence >0.1 µm^2^) and average areas of LC3 puncta in each LEC (each symbol represents one cell; *n*, numbers of cells analyzed; bars, mean ± SD; ***p <0.01*, *****p <0.0001*, Kruskal-Wallis test followed by Bonferroni post hoc test).

**
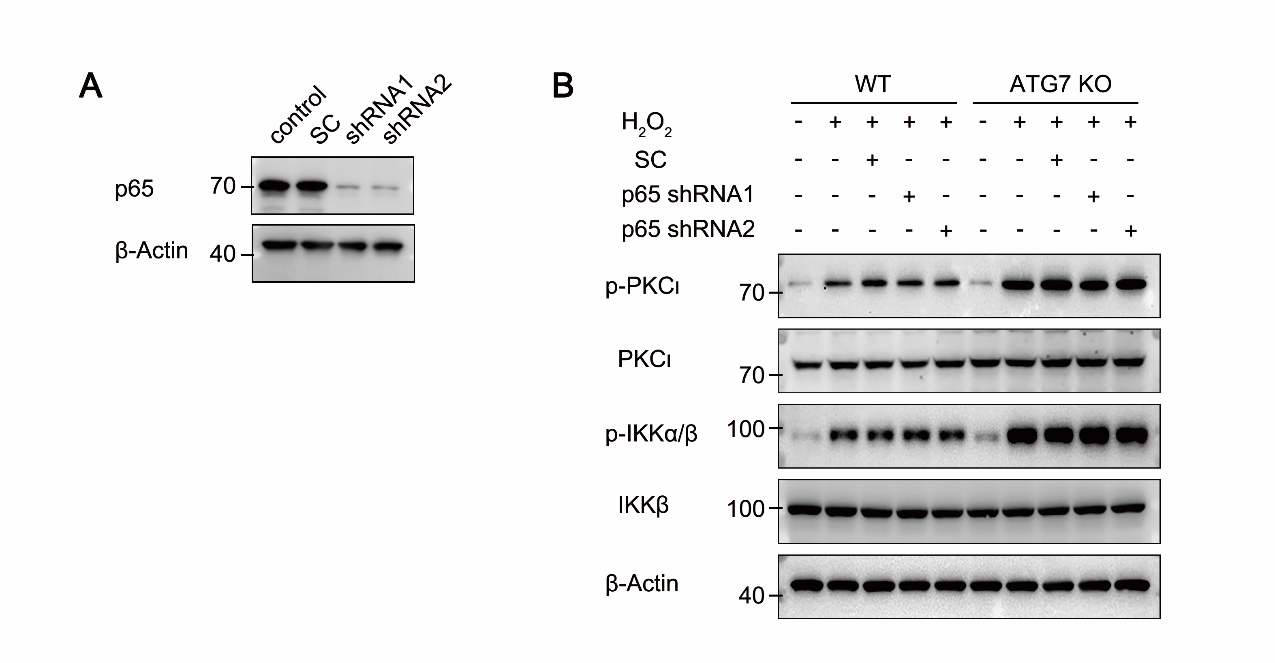
Figure S7. Knockdown of NF-κB p65 gene does not affect upstream signaling.** **(A)** Knockdown of p65 using lentiviral shRNA confirmed by immunoblot. Two independent interference sequences of p65 were used. **(B)** Immunoblots showing total and phosphorylated PKCι and IKKα/β levels in WT and ATG7 KO HLE-B3 cells before and after p65 RNA interference (RNAi) in the presence of 200 μM H_2_O_2_ for 2 h.

**
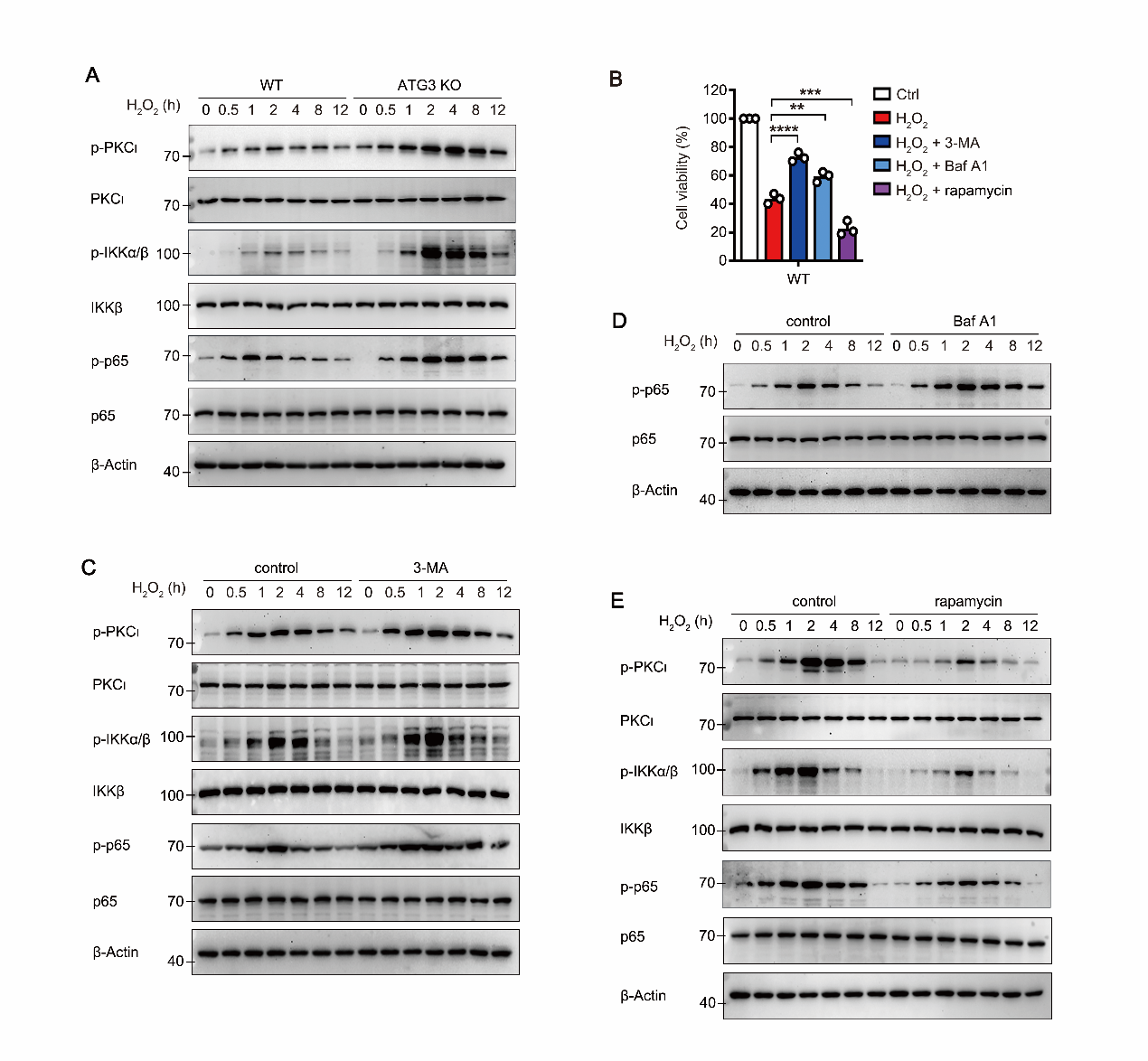
Figure S8. Autophagy regulates apoptosis and activation of the PKCι-IKK-NF-κB cascade in HLE-B3 cells. (A)** Immunoblots showing total and phosphorylated PKCι, IKKα/β, and p65 levels in WT and ATG3 KO HLE-B3 cells exposed to 200 μM H_2_O_2_ for indicated times. **(B)** Viability of WT HLE-B3 cells pretreated with 5 mM 3-MA for 3 h, 0.5 μM Baf A1 for 4 h, or 10 nM rapamycin for 4 h, followed by 200 μM H_2_O_2_ exposure for 24 h determined by CCK8 assay (mean ± SD; **p <0.01, ***p <0.001, ****p <0.0001, one-way ANOVA followed by Bonferroni post hoc test). **(C-E)** Total and phosphorylated PKCι, IKKα/β, and p65 levels in WT HLE-B3 cells pretreated with 5 mM 3-MA for 3 h (C), 0.5 μM Baf A1 for 4 h (D), or 10 nM rapamycin for 4 h (E) followed by 200 μM H_2_O_2_ treatment for indicated times.

**
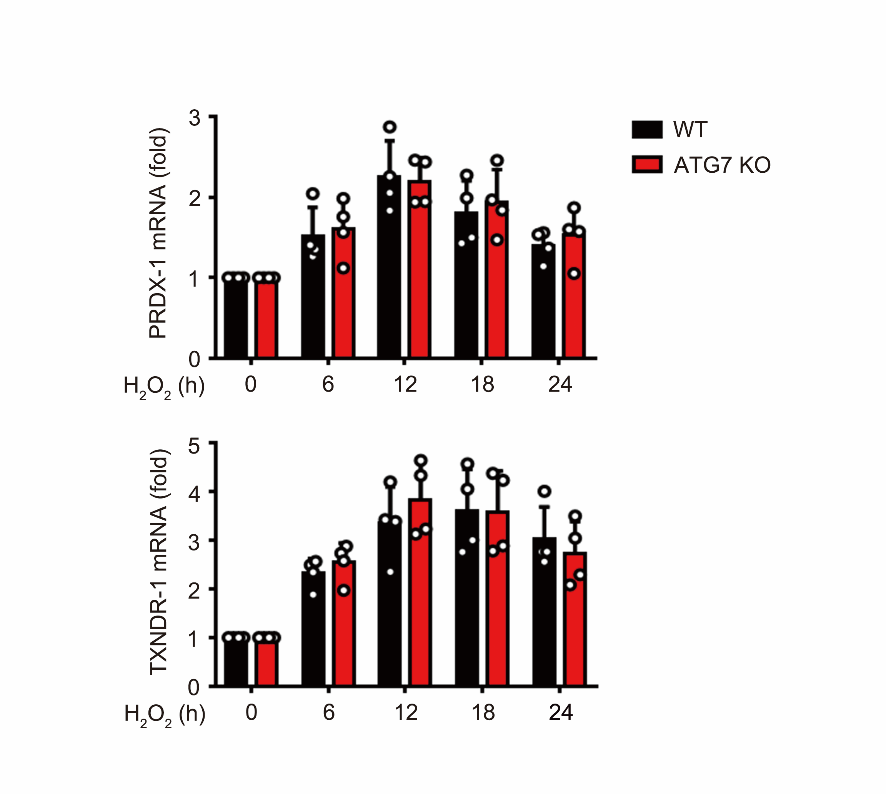
**

**Figure S9. Ablation of ATG7 not alter the expression of Nrf2 target genes in HLE-B3 cells under oxidative stress.** Relative mRNA levels of PRDX-1 and TXNDR-1 in WT and ATG7 KO HLE-B3 cells stimulated with 200 μM H_2_O_2_ for indicated times detected by real-time PCR (mRNA levels normalized by that in cells without H_2_O_2_ treatment).

**
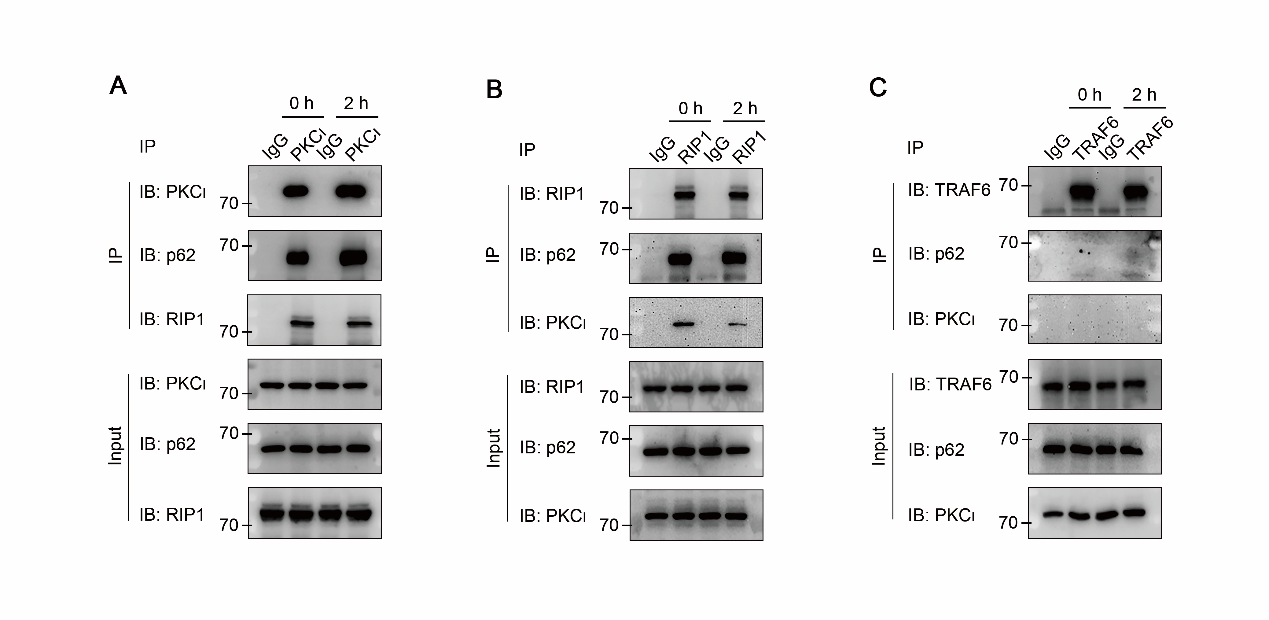
Figure S10. Confirmation of p62 partners under oxidative stress.** **(A-C)** Immunoblot analysis of precipitates before (Input, lower) and after (upper) affinity purification of PKCι (**A**), RIP1 (**B**), and TRAF6 (**C**) using corresponding antibodies, with or without 200 μM H_2_O_2_ for 2 h. Precipitates with anti-IgG antibody are negative controls.

**
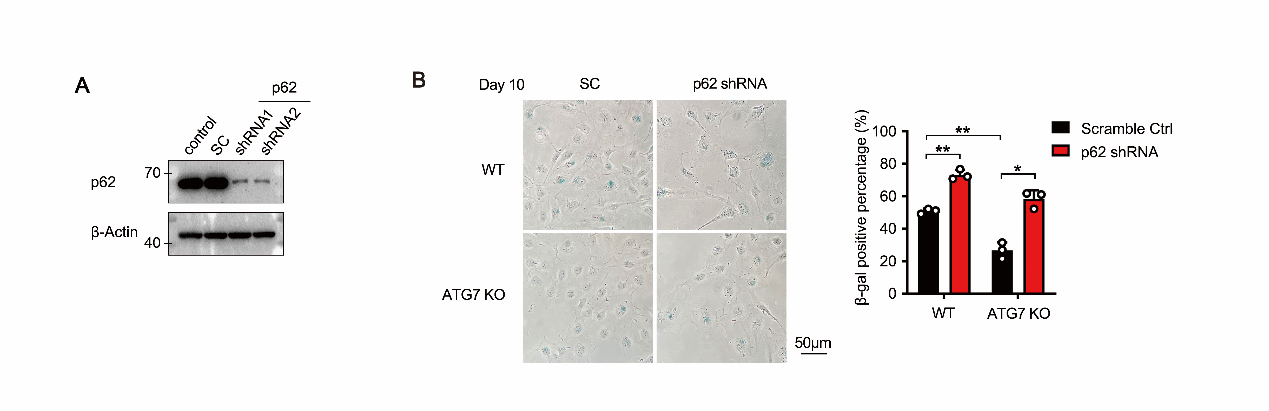
Figure S11. Knockdown of p62 alleviates cell senescence induced by oxidative stress.** **(A)** Knockdown of p62 using lentiviral shRNA confirmed by immunoblot. Two independent interference sequences were introduced. **(B)** β-gal staining of WT, ATG7 KO HLE-B3 cells (left panel) and quantification of the percentages of β-gal positive cells (right panel). Cells were knockdown of p62 or not, and followed 20 μM H_2_O_2_ treatment for 10 days. Data are mean ± SD from three random fields; **p <0.05, **p <0.01* (two-way ANOVA followed by Bonferroni post hoc test).

**
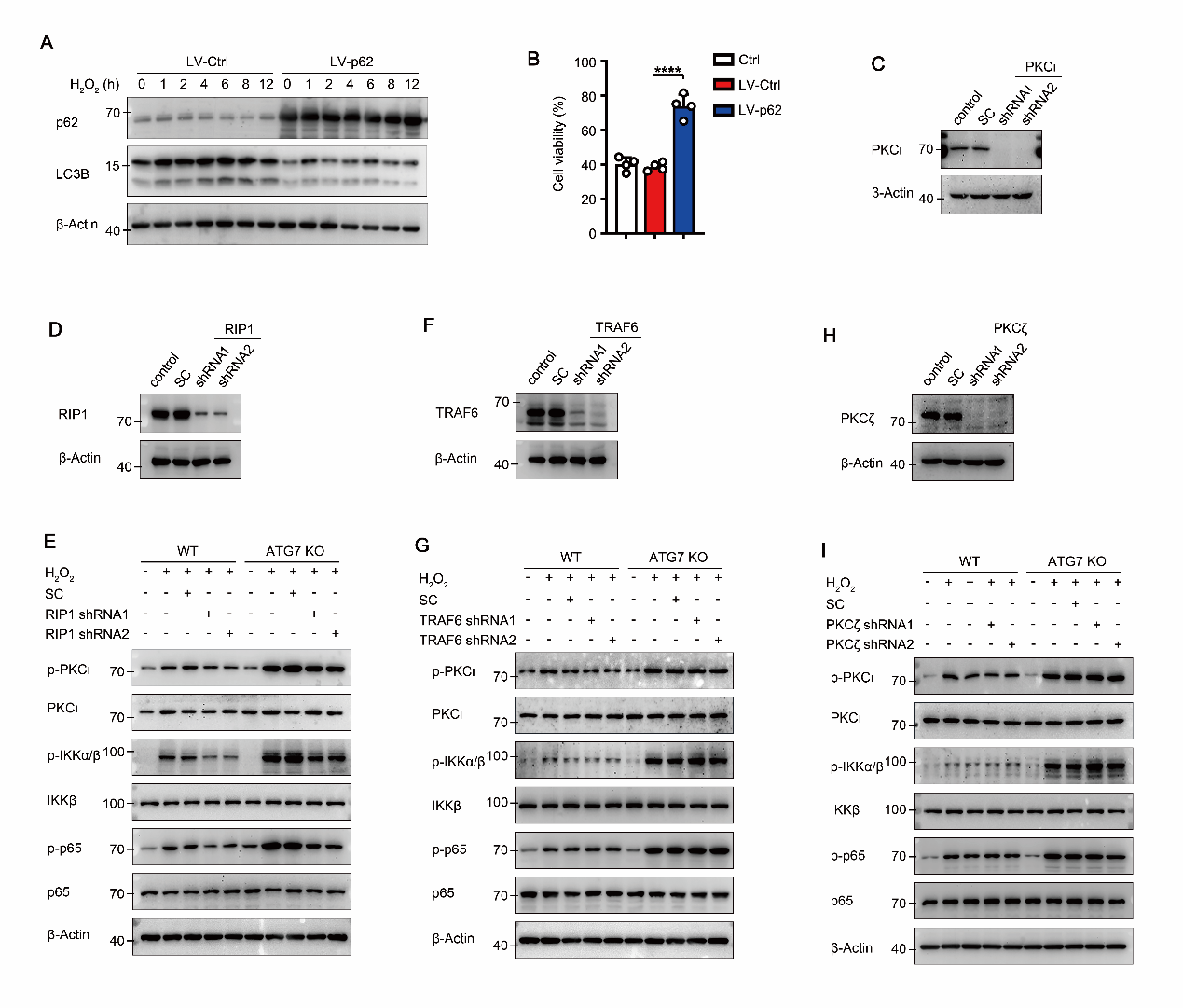
Figure S12. The p62-PKCι-RIP1 complex is essential for NF-κB activation under oxidative stress. (A)** Immunoblots showing p62, LC3B levels in HLE-B3 cells after p62 overexpression using lentiviral vector, in the presence of 200 μM H_2_O_2_ for indicated times. **(B)** Viability of WT HLE-B3 cells after p62 overexpression as determined by CCK8 assay (mean ± SD from four independent experiments; *****p <0.0001*, one-way ANOVA followed by Bonferroni post hoc test). **(C)** Knockdown of PKCι using lentiviral shRNA confirmed by immunoblot. Two independent interference sequences were introduced. **(D)** Knockdown of RIP1 using lentiviral shRNA confirmed by immunoblot. Two independent interference sequences were introduced. **(E)** Total and phosphorylated PKCι, IKKα/β, and p65 levels in WT and ATG7 KO HLE-B3 cells before and after RIP1 RNAi, in the presence of 200 μM H_2_O_2_ for 2 h. **(F)** Knockdown of TRAF6 using lentiviral shRNA confirmed by immunoblot. Two independent interference sequences were introduced. **(G)** Total and phosphorylated PKCι, IKKα/β, and p65 levels in WT and ATG7 KO HLE-B3 cells before and after TRAF6 RNAi, in the presence of 200 μM H_2_O_2_ for 2 h. **(H)** Knockdown of PKCζ using lentiviral shRNA confirmed by immunoblot. Two independent interference sequences were introduced. **(I)** Total and phosphorylated PKCι, IKKα/β and p65 level in WT and ATG7 KO HLE-B3 cells before and after PKCζ RNAi, in the presence of 200 μM H_2_O_2_ for 2 h.

**Table S1. Clinical data of senile cataract, transparent lens patients, and healthy donors**

|  | | **Senile cataract group** | | | | **Transparent lens group** | **Healthy donor group** | | |
| --- | --- | --- | --- | --- | --- | --- | --- | --- | --- |
| **Subgroups** | | **Mild** | **Moderate A** | **Moderate B** | **Severe** | **-** | **Young age** | **Middle age** | **Old age** |
| **Eyes (n)** | | 31 | 29 | 26 | 35 | 13 | 22 | 23 | 31 |
| **Age (y) (MD±SD, range)** | | 65.81±5.56  (52-77) | 73.86±7.53  (60-88) | 71.81±8.64  (54-84) | 79.17±7.40  (66-91) | 59.85±7.80  (46-72) | 25.59±2.79 (21-29) | 44.96±2.88 (40-49) | 64.65±3.18 (60-69) |
| **Sex ratio, Male/female**  **(%)*** | | 10/21  (32.26) | 13/16  (44.83) | 13/13  (50.00) | 14/21  (40.00) | 2/11  (15.38) | 13/9  (59.09) | 9/14  (39.13) | 14/17  (45.16) |
| **Cataract grading**  **(LOCS III)** | **C** | 1-3 | 1-3 | 4+ | 4+ | 0 | 0 | 0 | 0 |
|  | **N** | 1-3 | 4+ | 1-3 | 4+ | 0-1 | 0 | 0 | 0-1 |
|  | **P** | 0 | 0 | 0 | 0 | 0 | 0 | 0 | 0 |

* The distribution of sex ratio was comparable among all groups.

Senile cataract group: age-related cataract patients were categorized into four subgroups according to the degree of cataract; Mild, senile cataract with mild opacity of cortex or nucleus (grade 1-3); Moderate A: senile cataract with moderate opacity of cortex (grade 1-3) and nucleus (grade 4+); Moderate B: senile cataract with moderate opacity of cortex (grade 4+) and nucleus (grade 1-3); Severe: senile cataract with severe opacity of cortex (grade 4+) or nucleus (grade 4+).

Transparent lens group: patients who received transparent lens extraction surgery for the purpose of presbyopia correction.

Healthy donor group: Donor eyes with transparent lens for corneal transplantation were categorized into young, middle, and old age subgroups

LOCS III, Lens Opacities Classification System III; C, cortex; N, nucleus; P, posterior capsular.

**Table S2. shRNA sequences of different target genes**

| Target gene | shRNA sequence |
| --- | --- |
| p62 shRNA1 | 5’-GGATCCGAGTGTGAATTTCCT-3’ |
| p62 shRNA2 | 5’-CCGGAAGGTGAAACACGGACA-3’ |
| PKCι shRNA1 | 5’-AGTACTGTTGGTTCGATTAAA-3’ |
| PKCι shRNA2 | 5’- CCTGAAGAACATGCCAGAT-3’ |
| NF-κB p65 shRNA1 | 5’-CGGATTGAGGAGAAACGTAAA-3’ |
| NF-κB p65 shRNA2 | 5’-GGACATATGAGACCTTCAA-3’ |
| RIP1 shRNA1 | 5’-GGGAAGGTGTCTCTGTGTTTC-3’ |
| RIP1 shRNA2 | 5’-GCCGACATTTCCTGGCATTGA-3’ |
| PKCζ shRNA1 | 5’-CGCGTGATTGACCCTTTAACT-3’ |
| PKCζ shRNA2 | 5’-GCATGATGACGAGGATATTGA-3’ |
| TRAF6 shRNA1 | 5’-GGAATTTCCAGGAAACTATTC-3’ |
| TRAF6 shRNA2 | 5’-GCAACTTTGGAATGCATTTGA-3’ |
| Nrf2 shRNA1 | 5’-AGTTTGGGAGGAGCTATTAT-3’ |
| Nrf2 shRNA2 | 5’-CCGGCATTTCACTAAACAC-3’ |
| Scramble control | 5’-TTCTCCGAACGTGTCACGT-3’ |

**Table S3. Antibodies used for immunofluorescent staining, co-immunoprecipitation, and Western blot.**

| Primary Antibodies | Source | Company | Catalog No. | Dilution* |
| --- | --- | --- | --- | --- |
| TRAF6 (D21G3) | Rabbit mAb | Cell Signaling | 8028 | 1:1000  1:100 IP |
| NF-κB p65 (D14E12) | Rabbit mAb | Cell Signaling | 8242 | 1:1000  1:300 IF |
| Phospho-NF-κB p65  (Ser 536) (93H1) | Rabbit mAb | Cell Signaling | 3033 | 1:1000 |
| PKCι/λ (C83H11) | Rabbit mAb | Cell Signaling | 2998 | 1:1000 |
| PKCι/λ | Rabbit pAb | Abcam | ab227290 | 1:100 IF |
| PKCι/λ (H-12) | Mouse mAb | Santa Cruz  Biotechnology | sc-17837 | 1:30 IP |
| Phospho-PKC lambda/iota(Thr555, Thr563) | Rabbit mAb | Invitrogen | 700582 | 1:1000 |
| Caspase-3 (8G10) | Rabbit mAb | Cell Signaling | 9665 | 1:1000 |
| Caspase-8 (1C12) | Mouse mAb | Cell Signaling | 9746 | 1:1000 |
| Caspase-9 (C9) | Mouse mAb | Cell Signaling | 9508 | 1:1000 |
| Phospho-IKKα/β (Ser176/180) (16A6) | Rabbit mAb | Cell Signaling | 2697 | 1:1000 |
| XIAP | Rabbit mAb | Abcam | ab229050 | 1:1000 |
| Bcl-2 (D17C4) | Rabbit mAb | Cell Signaling | 3498 | 1:1000 |
| Bcl-xL (54H6) | Rabbit mAb | Cell Signaling | 2764 | 1:1000 |
| Bax (D2E11) | Rabbit mAb | Cell Signaling | 5023 | 1:1000 |
| ATG7 (EPR6251) | Rabbit mAb | Abcam | ab133528 | 1:1000 |
| ATG3 (EPR4801) | Rabbit mAb | Abcam | ab108251 | 1:1000 |
| IKKβ (2C8) | Rabbit mAb | Cell Signaling | 2370 | 1:1000 |
| SQSTM1/p62 | Mouse mAb | Abcam | ab56416 | 1:1000  1:300 IF |
| SQSTM1/p62 | Rabbit pAb | Proteintech | 18420-1-AP | 1:100 IP |
| PKC zeta | Rabbit pAb | Invitrogen | PA5-78129 | 1:1000 |
| RIP (D94C12) | Rabbit mAb | Cell Signaling | 3493 | 1:1000  1:100 IP |
| LC3B | Rabbit pAb | Cell Signaling | 2775 | 1:1000 |
| LC3 | Rabbit pAb | MBL | PM036 | 1:200 IF |
| IκBα (L35A5) | Mouse mAb | Cell Signaling | 4814 | 1:1000 |
| Phospho-IκBα (Ser32) (14D4) | Rabbit mAb | Cell Signaling | 2859 | 1:1000 |
| Histone H3 (3H1) | Rabbit mAb | Cell Signaling | 9717 | 1:1000 |
| KEAP1 (EPR22664-26) | Rabbit mAb | Abcam | ab227828 | 1:1000 |
| Nrf2 (EP1808Y) | Rabbit mAb | Abcam | ab62352 | 1:1000 |
| Nrf2 (phospho S40) (EP1809Y) | Rabbit mAb | Abcam | ab76026 | 1:1000 |
| β-Actin(8H10D10) | Mouse mAb | Cell Signaling | 3700 | 1:1000 |
| Secondary Antibodies | Source | Company | Catalog No. | Dilution |
| Goat Anti-Mouse IgG (H&L) (HRP) | Goat pAb | Abcam | ab205719 | 1:2000 |
| Goat anti-Rabbit IgG (H&L) (HRP) | Goat pAb | Abcam | ab205718 | 1:2000 |
| Goat anti-Rabbit IgG (H&L) (Alexa Fluor® 488) | Goat pAb | Abcam | ab150077 | 1:1000 IF |
| Goat anti-Mouse IgG (H&L) (Alexa Fluor® 488) | Goat pAb | Abcam | ab150113 | 1:1000 IF |
| Goat anti-Rabbit IgG (H&L) (Alexa Fluor® 555) | Goat pAb | Abcam | ab150078 | 1:1000 IF |
| Goat anti-Mouse IgG (H&L) (Alexa Fluor® 555) | Goat pAb | Abcam | ab150114 | 1:1000 IF |
| Goat anti-Rabbit IgG (H&L) (Alexa Fluor® 647) | Goat pAb | Invitrogen | A-21245 | 1:1000 IF |

*Dilution ratio of antibodies without following IP or IF was used for Western bolt.

**Table S4. Primer sequences for real-time PCR**

| Gene | Species | Forward | Reverse |
| --- | --- | --- | --- |
| BCL2L1 | Human | 5’-GACTGAATCGGAGATGGAGACC-3’ | 5’-GCAGTTCAAACTCGTCGCCT-3’ |
| BCL2 | Human | 5’-GGTGGGGTCATGTGTGTGG-3’ | 5’-CGGTTCAGGTACTCAGTCATCC-3’ |
| XIAP | Human | 5’-ACCGTGCGGTGCTTTAGTT-3’ | 5’-TGCGTGGCACTATTTTCAAGATA-3’ |
| BAX | Human | 5’-CCCGAGAGGTCTTTTTCCGAG-3’ | 5’-CCAGCCCATGATGGTTCTGAT-3’ |
| p62 | Human | 5’-AAGCCGGGTGGGAATGTTG-3’ | 5’-CCTGAACAGTTATCCGACTCCAT-3’ |
| TXNRD1 | Human | 5’-ATATGGCAAGAAGGTGATGGTCC-3’ | 5’-GGGCTTGTCCTAACAAAGCTG-3’ |
| PRDX1 | Human | 5’-CCACGGAGATCATTGCTTTCA-3’ | 5’-AGGTGTATTGACCCATGCTAGAT-3’ |
| β-actin | Human | 5’-CATGTACGTTGCTATCCAGGC-3’ | 5’-CTCCTTAATGTCACGCACGAT-3’ |
