# Supplementary material for: Autophagy facilitates age-related cell apoptosis—a new insight from senile cataract
Source: Cell Death Dis. 2022 Jan 10;13(1):37. doi: 10.1038/s41419-021-04489-8 (PMC8748728; doi:10.1038/s41419-021-04489-8)
Supplement: Supplementary file 3 — Declaration of contributions [file 41419_2021_4489_MOESM3_ESM.pdf]

DECLARATION OF CONTRIBUTIONS TO ARTICLE

ADMC

Manuscript Number:

Journal Name:

Cell Death & Differentiation (the 'Journal')

Proposed Title of the Contribution:

Autophagy facilitates age-related cell apoptosis - a new insight from senile cataract (the 'Contribution)

Author(s):

Jiani Huang, Wangshu Yu, Qin He, Xiaoying He, Ming Yang, Wei Chen, Wei Han (the 'Authors')

For all *CDD* articles, each person named as an author in the published version must be able to show he or she has contributed substantially to the article.

Authorship credit should be based on 1) substantial contributions to conception and design, acquisition of data, or analysis and interpretation of data; 2) drafting the article or revising it critically for important intellectual content; and 3) final approval of the version to be published. Authors should meet conditions 1, 2 and 3.

Any person who cannot be shown to have made a substantial contribution to the article cannot be listed as an author in the final version. The name of any person who is deemed to have made a minor contribution can, however, appear in the Acknowledgments section of the article.

Please complete the table below to indicate the contributions of all named authors to the manuscript.

Author Full Name:	Specification of Contribution to the Manuscript:
Wei Han	designed the study, wrote the manuscript and approved the final version
Wei Chen	designed the study, wrote the manuscript and approved the final version
Jiani Huang	performed research, collected the data, analyzed and interpreted the data, performed the statistical analysis, revised the manuscript and approved the final version
Wangshu Yu	performed research, collected the data, analyzed and interpreted the data, performed the statistical analysis, revised the manuscript and approved the final version
Qin He	performed research, collected the data, analyzed and interpreted the data, performed the statistical analysis, revised the manuscript and approved the final version
Xiaoying He	performed research, collected the data, revised the manuscript and approved the final version
Ming Yang	performed research, collected the data, revised the manuscript and approved the final version

Please complete the table below to indicate the contributions of all named authors to the figures.

Figure 1:

JH generated immunofluorescence data and labelled the image (panel A). QH and JH performed the statistical analysis (panel B). JH generated electron micrograph data and labelled the image (panel C). JH assembled the figure.

Figure 2:

JH generated the data and prepared panel A, B and F. JH and XH generated the western blot data and labelled the image (panel D and E). WY generated the data and prepared panel C. JH generated immunofluorescence data and labelled the image (panel H). JH performed the statistical analysis (panel G). QH and WY performed the statistical analysis (panel I and J). JH assembled the figure.

Figure 3:

WY generated the flow cytometry (panel A, B and F). XH generated the data and prepared panel C-D. QH generated the cell viability analysis and prepared panel E. QH performed the statistical analysis. JH assembled the figure.

Figure 4:

WY prepared the sample, analyzed the RNA sequencing data and prepared panel A and B. WY and MY generated the data and prepared panel C. JH and WY generated the western blot data and labelled the image (panel D, E and F). XH completed the lentivirus packaging. JH generated the cell viability analysis and prepared panel G. WY generated immunofluorescence data and labelled the image (panel H). QH performed the statistical analysis. JH assembled the figure.

Figure 5:

JH generated immunofluorescence data and labelled the image (panel A). QH performed the statistical analysis (panel B). JH and WY generated the western blot data and labelled the image (panel C and F). MY generated the data and prepared panel D. WY prepared the sample, analyzed the RNA sequencing data and prepared panel E. XH completed the lentivirus packaging. JH generated the cell viability analysis and prepared panel G. JH assembled the figure.

Figure 6:

JH and QH generated the western blot data and labelled the image (panel A, D, F, G and H). XH and JH completed the lentivirus packaging, generated the cell viability analysis and prepared panel E. WY generated immunofluorescence data and labelled the image (panel B and C). QH performed the statistical analysis. JH assembled the figure.

Figure 7: WY generated immunofluorescence data and labelled the image (panel A and D). WY generated the data and prepared panel B, C and E-G. QH performed the statistical analysis. JH assembled the figure.

Figure 8: QH drew the diagram and assembled the figure.

Signed for and on behalf of the Author(s):	Print Name:	Date:
Hanwei Wei Chen	Wei Han, Wei Chen	May 30, 2021
